# Computational Identification of the Paralogs and Orthologs of Human Cytochrome P450 Superfamily and the Implication in Drug Discovery

**DOI:** 10.3390/ijms17071020

**Published:** 2016-06-28

**Authors:** Shu-Ting Pan, Danfeng Xue, Zhi-Ling Li, Zhi-Wei Zhou, Zhi-Xu He, Yinxue Yang, Tianxin Yang, Jia-Xuan Qiu, Shu-Feng Zhou

**Affiliations:** 1 Department of Oral and Maxillofacial Surgery, the First Affiliated Hospital of Nanchang University, Nanchang 330003, China; panshuting314@126.com (S.-T.P.); danfengxue@163.com (D.X.); 2 Department of Pharmacy, Shanghai Children’s Hospital, Shanghai Jiao Tong University, Shanghai 200040, China; lizhiling22@163.com; 3 Department of Pharmaceutical Sciences, School of Pharmacy, Texas Tech University Health Sciences Center, Amarillo, TX 79106, USA; zhiwei.zhou@ttuhsc.edu; 4 Guizhou Provincial Key Laboratory for Regenerative Medicine, Stem Cell and Tissue Engineering Research Center & Sino-US Joint Laboratory for Medical Sciences, Guizhou Medical University, Guiyang 550004, China; hzx@gmc.edu.cn; 5 Department of Colorectal Surgery, General Hospital of Ningxia Medical University, Yinchuan 750004, China; nyfyyyx@126.com; 6 Department of Internal Medicine, University of Utah and Salt Lake Veterans Affairs Medical Center, Salt Lake City, UT 84132, USA; tianxin.yang@hsc.utah.edu; 7 Department of Chemical and Pharmaceutical Engineering, College of Chemical Engineering, Huaqiao University, Xiamen 361021, Fujian, China

**Keywords:** human *CYP*, drug metabolism, paralog, homolog, ortholog, comparative genomics, bioinformatics

## Abstract

The human cytochrome P450 (CYP) superfamily consisting of 57 functional genes is the most important group of Phase I drug metabolizing enzymes that oxidize a large number of xenobiotics and endogenous compounds, including therapeutic drugs and environmental toxicants. The *CYP* superfamily has been shown to expand itself through gene duplication, and some of them become pseudogenes due to gene mutations. Orthologs and paralogs are homologous genes resulting from speciation or duplication, respectively. To explore the evolutionary and functional relationships of human *CYPs*, we conducted this bioinformatic study to identify their corresponding paralogs, homologs, and orthologs. The functional implications and implications in drug discovery and evolutionary biology were then discussed. GeneCards and Ensembl were used to identify the paralogs of human *CYPs*. We have used a panel of online databases to identify the orthologs of human *CYP* genes: NCBI, Ensembl Compara, GeneCards, OMA (“Orthologous MAtrix”) Browser, PATHER, TreeFam, EggNOG, and Roundup. The results show that each human *CYP* has various numbers of paralogs and orthologs using GeneCards and Ensembl. For example, the paralogs of *CYP2A6* include *CYP2A7*, *2A13*, *2B6*, *2C8*, *2C9*, *2C18*, *2C19*, *2D6*, *2E1*, *2F1*, *2J2*, *2R1*, *2S1*, *2U1*, and *2W1*; *CYP11A1* has 6 paralogs including *CYP11B1*, *11B2*, *24A1*, *27A1*, *27B1*, and *27C1*; *CYP51A1* has only three paralogs: *CYP26A1*, *26B1*, and *26C1*; while *CYP20A1* has no paralog. The majority of human *CYPs* are well conserved from plants, amphibians, fishes, or mammals to humans due to their important functions in physiology and xenobiotic disposition. The data from different approaches are also cross-validated and validated when experimental data are available. These findings facilitate our understanding of the evolutionary relationships and functional implications of the human *CYP* superfamily in drug discovery.

## 1. Introduction

The cytochrome P450s (CYPs) are a large heme-containing enzyme superfamily with a large number of members. They are found across all organisms from animals, plants, fungi, protists, bacteria, and Archaea to even viruses [1,2,3]. The CYP enzymes were first reported in 1958 since they displayed a Soret peak at 450 nm (therefore known as “P450” or “Pigment at 450 nm”). This featured peak is generated via a thiolate anion which serves as the fifth binding ligand to the heme moiety, and this unique peak is only found in P450s, chloroperoxidases, nitric oxide synthases, and protein H450 (all belong to the hemoprotein superfamily) [4,5,6]. CYPs are responsible for metabolizing numerous exogenous and endogenous compounds, including steroids, fatty acids, retinoids, clinical drugs, vitamins, procarcinogens/promutagens, and environmental compounds [7,8,9]. To date, at least 39,417 CYPs from 236 species have been reported, with 22,675 CYPs from 129 species of fungi (57.53%) and 16,742 CYPs from non-fungal species (42.47%). The *CYP* superfamily comprises a family, subfamilies, and individual members. As hemoproteins, CYPs often catalyze various modes of oxidative reactions such as hydroxylation, sulphoxidation, demethylation and dealkylation, deamination, dehalogenation, epoxidation, and peroxidation of their substrates [10]. In addition to these classical oxidative reactions, CYPs also catalyze some uncommon oxidative or reductive reactions of certain substrates such as oxidative cleavage of carboxylic acid esters, desaturation, 1- and 2-electron reductions, 1-electron oxidation, rearrangements of oxidized eicosanoids, deformylation of aldehydes, ring formation and aldoxime dehydration, etc.

The majority of CYPs are mixed function oxidases or monooxygenases, and electrons needed for reduction of the heme, and subsequently the oxygen substrates are provided by special partners [11,12]. For example, all microsomal CYPs are able to transfer electrons from nicotinamide adenine dinucleotide phosphate (NADPH) as the donor via cytochrome P450 reductase while cytochrome *b*_5_ is a ubiquitous electron carrier which is reduced by cytochrome *b*_5_ reductase (also called methemoglobin reductase) [11,12,13,14,15]. On the other hand, mitochondrial CYPs employ adrenodoxin reductase and adrenodoxin to transfer electrons from NADPH to the enzyme [16]. However, CYP5A1 (called thromboxane X_2_ synthase, TBXAS1), CYP8A1 (called prostacyclin H_2_ synthase, PTGIS), and CYP74A (called allene oxide synthase) do not require a protein partner for their enzymatic catalytic reactions [17]. The catalytic cycle by CYPs is similar and complicated, with the heme prosthetic group acting as the catalytic center of the enzyme. Upon initiation of the CYP-mediated catalytic reaction, a hypervalent oxo-iron protoporphyrin IX radical cation is produced, facilitating subsequent insertion of the iron-bound oxygen into a substrate bond. When the thiolate side chain of a conserved Cys attaches to the iron molecule opposite to the bound oxygen, the substrate molecule moves in and binds with multiple residues in a cavity (active site) above the heme moiety near the reactive radical cation. Although CYPs share a catalytic mechanism, they show remarkable differences in substrate specificity, regio-, and stereo-selectivity of metabolic reaction, and inhibition by molecules [18].

The gain and loss events are not evenly distributed among the *CYP* genes and *CYP* genes and pseudogenes are often present as clusters in the genome of organisms. Continuing and consecutive tandem gene duplication including unequal crossover, transposable element-induced non-allelic recombination, chromosome duplication, and retroposition may result in relatively large *CYP* clusters on some chromosomes, which often represent remarkable landmarks of the CYPomes in organisms. For instance, the mouse cluster of *Cyp2* genes on chromosome 7 carries 12 functional genes and 10 pseudogenes [19]. The largest ancestral *CYP6A* cluster in fruit flies contains 8 *CYP6A* genes and *CYP317A1* at 51D region of the right arm of chromosome 2 [20]. Among these genes, *CYP6A17*-*21* is coordinately regulated by the circadian rhythm [21]. In the malaria vector *Anopheles gambiae*, the largest *CYP* gene cluster contains 14 *CYP6* genes and another cluster carries 12 *CYP325* genes and 2 pseudogenes [22]. In mosquito genomes, there are 16 *CYP* gene clusters consisting of ≥4 genes while 22 *CYP* genes are present as singletons [22]. Rats contain 14 *Cyp2* genes and 4 pseudogenes and humans carry 6 *CYP2* genes and 7 pseudogenes in the same cluster. The human *CYP2C* cluster located on chromosome 10 consists of only 4 functional genes, but it has expanded to 15 functional genes on chromosome 9 (*Cyp2c29*, *2c37-2c40*, *2c44*, *2c50*, *2c54*, *2c55*, and *2c65-2c70*) in mice. In the rat genome, the *Cyp2c* cluster on chromosome 1q contains 13 functional genes, including *Cyp2c6/2c37*, *2c7/2c39*, *2c11*, *2c12/2c40*, *2c13/2c38*, *2c22-2c24*, *2c26*, *2c29*, *2c66*, *2c79/2c65*, and *2c80/2c55*.

Human *CYP* members play an important role in oxidizing a number of endogenous and exogenous compounds, including >80% of clinically used drugs and physiologically important substrates such as retinoids and fatty acids [8,23]. According to the current human genome GRCh38.p6 assembly (Genome Reference Consortium Human Build 38 patch release 6) released on 23 December 2015, there are 57 functional *CYP* genes and 58 pseudogenes distributed into at least 18 families and 43 subfamilies. *CYP1*, *2*, *3*, and *4* families play a major role in the oxidative metabolism of most drugs while other CYP families are responsible for the metabolism of several groups of important endogenous compounds including steroids, fatty acids, and retinoids [7,8,24,25,26]. The relative abundance of human hepatic CYPs is as: CYP1A1 (<1%), 1A2 (4.4%–16.3%), 2A6 (3.5%–14%), 2B6 (1.7%–5.3%), 2C8 (~7.5%), 2C9 (4.5%–29%), 2C19 (0.9%–3.8%), 2D6 (1.3%–4.3%), 2E1 (5.5%–16.5%), 2J2 (<1%), 3A4 (14.5%–37%), and 3A5 (~1%) [27]. A study has shown that 66% of the 110 drugs examined are metabolized by one or more CYPs, and 44% of these drugs are metabolized by CYP3A4, 41% by 2D6, 26% by 2C19, 9% by 1A2, and 4% by 2C9 [28]. As functionally important enzymes, *CYPs* have been well studied in humans, mice, and rats, but data are limited in other species. It is important to identify suitable animal models for CYP studies in drug discovery and toxicological studies since the data can be readily extrapolated to humans with reasonable accuracy, although there are marked differences in the anatomical, biochemical, and physiological characteristics between these animals and human beings.

A multigene family or superfamily may arise from gene duplication and amplification, expression of overlapping genes, exon shuffling, programmed frame shifting, alternative RNA splicing, and gene sharing (also called “protein moonlighting” where the same protein fulfils at least two strikingly different functions) [29,30,31,32,33]. Exon shuffling is a molecular process through which two or more exons from distinct genes are recombined ectopically, or the same exon can be duplicated to produce a novel intron-exon structure. This process may be transposon-mediated, or it can arise from crossover during meiosis and recombination between non-homologous or short homologous DNA sequences. Gene duplication/amplification results from ectopic homologous recombination, aneuploidy, whole genome duplication, retrotransposition, and replication slippage (also called slipped-strand mispairing resulting in either a trinucleotide or dinucleotide expansion or contraction during DNA replication) [34,35]. Based on currently available molecular genetic evidence from comparative genomic and phylogenetic studies, it is speculated that modern *CYPs* stem from an ancestral gene that was present ~3.5 billion years ago before eukaryotes appeared and an oxygen-rich atmosphere existed [1,3,36,37]. The *CYP* superfamily may have undertaken repetitive rounds of expansion via gene duplication events [1,3,36,37,38]. The first gene expansion might occur ~1.5 billion years ago, resulting in several *CYP* families such as *CYP4* and *11* that primarily metabolized endogenous cholesterol and fatty acids. Another *CYP* gene expansion might occur ~900 million year ago (Mya), giving rise to several *CYP* families such as *CYP19*, *21*, and *27* that are responsible for steroid biosynthesis. Around 400 Mya, there was a striking expansion of *CYP* genes, resulting in several *CYP* families (e.g., *CYP2*, *3*, *4*, and *6*) that participated in xenobiotic metabolism. These *CYP* genes are rapidly evolving since the cells need protection from the damage when exposed to increasing toxic xenobiotics [3,36,37]. A well-defined distinction between orthologs and paralogs is important for the robust evolutionary classification of *CYP* genes and it is also critical for making reliable functional annotation of newly sequenced genomes from various organisms. Orthologs and paralogs are homologous genes arising from speciation or duplication event, respectively. Orthologs are generally considered to preserve equivalent or similar functions across different organisms [39,40]. A function-oriented group of orthologous genes comprises orthologs that retain the same functions in different species and recently evolved paralogous genes that have the same biological activities (also called “in-paralogs”) [41]. Construction of orthologous groups is essential when conducting phylogenetic and comparative genomic studies and transferring verified annotations to newly sequenced genomes in other species [42]. In addition, identification of orthologous genes across different species can facilitate delineation of the gene genealogy to probe the driving forces and mechanisms through which to generate orthologous genes. On the other hand, pseudogenes are produced during evolution of genomes, which are characterized by a combination of homology to a known functional gene and lack of functionality (inability to code a protein) due to premature stop codons and frameshifts [43,44,45]. In human genome GRCh38.p5 assembly, there are 14,453 pseudogenes including 58 *CYP* pseudogenes. Vertebrate genomes typically contain 57–120 *CYP* genes. For example, Rhesus monkesy have 114 *CYP* genes, pigs contain 58 *CYP* genes, dogs embrace 48 *CYP* genes, mice carry 102 *Cyp* genes and 88 pseudogenes, rats retain 89 *Cyp* genes and 79 pseudogenes, and zebrafish carries 81 *Cyp* genes. *Caenorhabditis elegans* contains 83 *Cyp* genes and fruit flies retain 84 *CYP* genes and 6 pseudogenes. *Oryza sativa* (rice) carries 457 *Cyp* genes and *Arabidopsis thaliana* contains 272 *Cyp* genes. To further explore the evolutionary and functional relationships of human *CYPs*, we conducted this study to identify their corresponding paralogs, homologs, and orthologs. The data from different approaches were cross-validated and validated when experimental data are available. Finally, the implications in drug discovery and toxicological studies were then briefly discussed.

## 2. Results

### 2.1. Alignment of 57 Human CYPs

**Figure 1:**
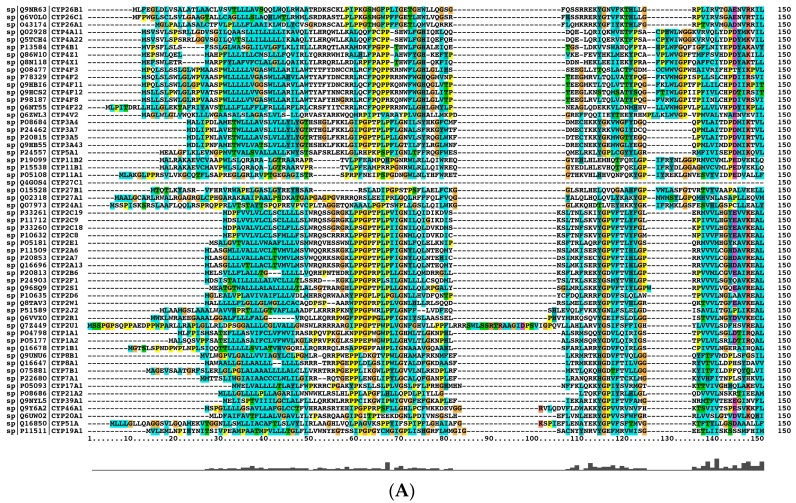

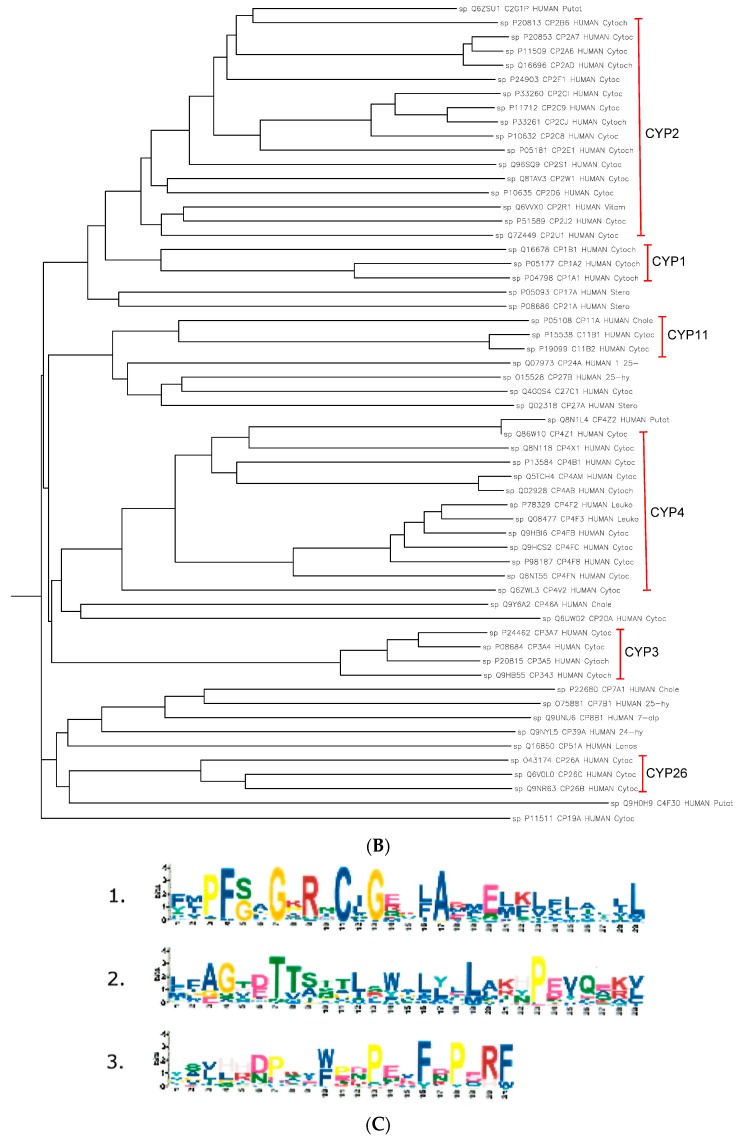
(**A**) Alignment of 57 human CYP proteins which are retrieved from Swiss-Prot. Multiple sequence alignment of human CYPs is carried out using Clustal W v2.0; (**B**) The phylogenic tree of human CYPs which can infer the evolutionary relationships among human CYPs; (**C**) MEME (Multiple EM for Motif Elicitation) version 4.10.1 is employed to identify important conserved motifs present in human CYP proteins.

The alignment of the 57 human CYP proteins has identified four conserved amino acids, namely Glu242, Arg245, Phe310, and Cys316 (Figure 1A). Phe310 and Cys316 are located near the heme-binding region and so play an important role in the catalysis. Cys316 is near the iron ion in the heme-binding region, acting as a critical thiolate ligand in the active site of CYPs. Glu242 and Arg245 located about 80 amino acids upstream from the proximal Cys316 may also play a role in enzymatic catalysis.

Next, we constructed the phylogenic tree of human *CYP* genes (Figure 1B), in which each monophyletic group was reinforced by a relatively high bootstrap value. In this phylogenic tree, an ancestral gene of *CYP17A1* and *21A1* seemed to be duplicated, resulting in the ancestor of the *CYP1* and *2* families. *CYP3* and *5* families appeared to have a common ancestor, while *CYP3* family appeared to be the ancestors of *CYP4* family. The ancestors of the *CYP1*, *2*, *3*, *4*, *11*, and *26* families were generated by gene duplication events of *CYP11A1* and *11B1*. Furthermore, CYP4V2 was formed from the duplication of the ancestors of *CYP46A* and *22A*, which was then duplicated to generate the whole *CYP4* family.

Finally, we searched the conserved motifs in the human CYP proteins by sequence alignment (Figure 1C). We have identified at least 3 well conserved motifs in human CYPs: “FXXGXRXCXG” located in the heme-binding domain, “AGXDTT”, and “EXXR” located in helix K in C-terminal. The terminal Thr residue in “AGXDTT” is involved in the formation of the enzyme’s critical oxygen-binding pocket, and “EXXR” can interact with the loop of ~14 amino acid C-terminal. These motifs are functionally essential for the enzymatic activity.

### 2.2. The Paralogs, Homologs and Orthologs of CYP1A1, 1B1, 1A2, 17A1, and 21A2

**Figure 2:**
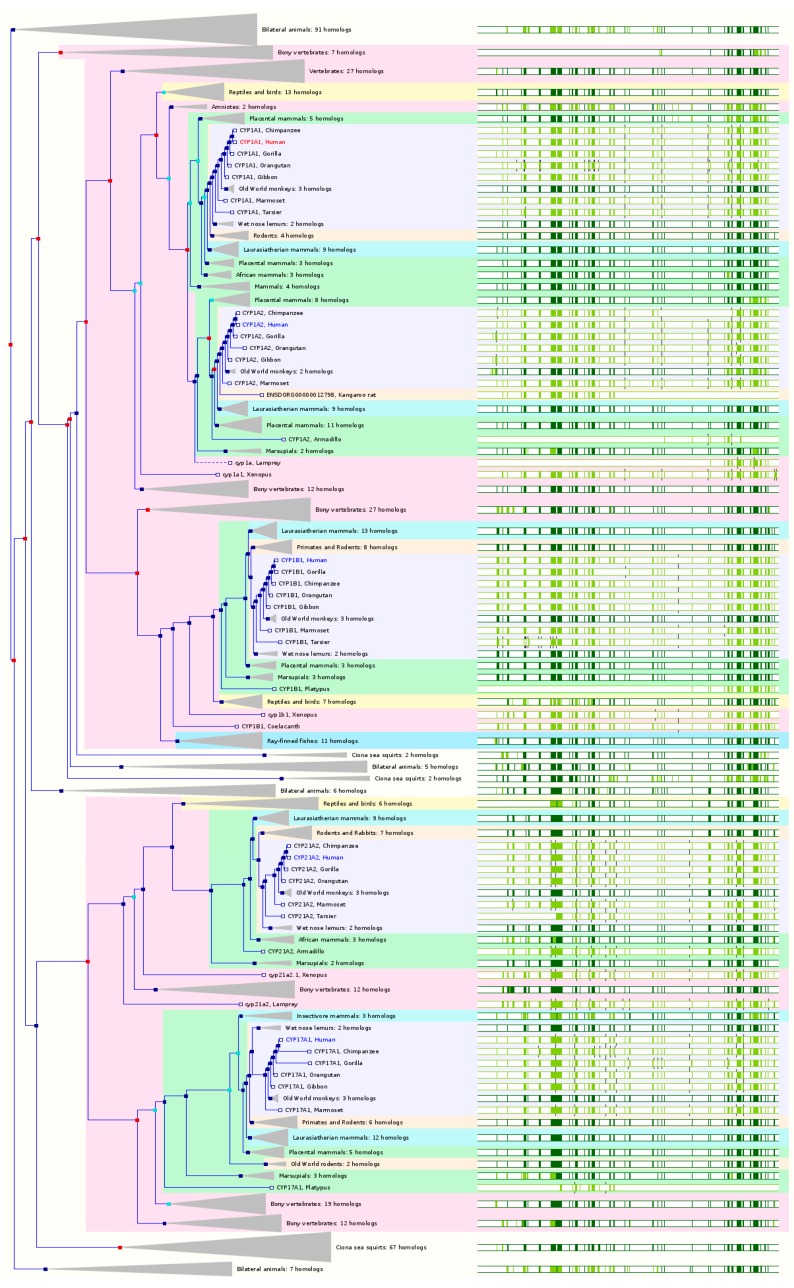

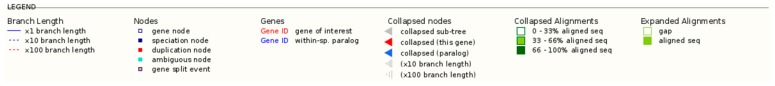
Gene tree for human *CYP1A1*, *1A2*, *1B1*, *17A1*, and *21A2* built using Ensembl 84. These five genes are paralogs to each other derived from the same ancestral gene via duplication events. The gene tree includes a total of 537 genes from various species. The total number of speciation nodes is 370, and the number of duplication is 143. The number of ambiguous nodes is 21, and the number of gene split events is 2.

The human genome contains three functional *CYP1* genes including *CYP1A1*, *1A2*, and *1B1* and one pseudogene *CYP1D1P/1A8P*. *CYP1D1* became a pseudogene in human and cattle due to five nonsense mutations in the putative coding region; however, several other mammals including chimpanzee, Rhesus monkey, and cynomolgus monkey possess a functional *CYP1D1*. *CYP1D1* is also conserved in the zebrafish, frog, *Magnaporthe oryzae* (*M. oryzae*), and rice. In cynomolgus monkey, *CYP1D1* is 95% identical to human *CYP1D1P* sequence and is mainly expressed in the liver, kidney, and jejunum. Cynomolgus monkey CYP1D1 heterologously expressed in *E. coli* catalyzes ethoxyresorufin *O*-deethylation and caffeine 8-hydroxylation, which human CYP1A1/1A2 also catalyze. Based on both GeneCards 4.1.1 and Ensembl 84, *CYP17A1* and *21A2* are the paralogs of *CYP1A1*, *1A2* and *1B1* (Figure 2 and Table 1).

CYP1A1 is primarily distributed in extrahepatic tissues, but it is involved in the metabolism of a number of clinical drugs (e.g., acetaminophen, caffeine, propranolol, and theophylline), certain procarcinogens (e.g., polycyclic aromatic hydrocarbons and aristolochic acid), toxicants and environmental compounds, and some endogenous substrates such as arachidonic acid (AA) and 17β-estradiol. The gene is highly inducible and mainly expressed in fetal liver. It has a low level in adult liver, lung, skin, intestine, skin, and gallbladder. In NCBI HomoloGene 68, *CYP1A1* has 14 homologenes in 11 species, including chimpanzee, Rhesus monkey, dog, mouse, rat, Xenopus, zebrafish, etc. Based on NCBI Annotation Pipeline, 98 organisms have orthologs with *CYP1A1* (Table S2). These include non-human primates, rodents, even-toed ungulates and whales, other mammals, birds, fishes, other vertebrates, etc. In Ensembl 84, *CYP1A1* has 71 orthologs from 63 species of chordates including 11 species of non-human primates, 7 species of rodents, 12 species of Laurasiatheria, 35 species of placental mammals, 7 species of Sauropsida, and 11 species of fishes (Figure 2 and Table S3). In GeneCards version 4.1.1, *CYP1A1* has orthologs in 15 species including chimpanzee, cattle, dog, mouse, rat, etc. (Table S4). Similar results with remarkable differences are observed with OMA, PANTHER, and EggNOG (Tables S5–S7).

CYP1A2 is a hepatic enzyme that oxidizes drugs (e.g., caffeine, clozapine, tacrine, theophylline, propranolol, and acetaminophen), procarcinogens and environmental compounds (e.g., benzopyrene, aflatoxin B_1_, and nicotine), and several groups of endogenous substrates (e.g., steroids and AA). Hepatic CYP1A2 is highly inducible by proton pump inhibitors such as omeprazole, smoking, polyamine hydrocarbons from grilled meats, and dietary cruciferous vegetables. Furafylline is a selective and potent inhibitor for human CYP1A2, but it is a weak inhibitor for mouse, rat and dog CYP1A2/1a2 and no inhibitory effect on monkey CYP1A2. In NCBI HomoloGene 68, *CYP1A2* has 7 homologenes in 7 species, including chimpanzee, dog, cattle, mouse, rat, etc. Based on NCBI Annotation Pipeline, 69 organisms have orthologs with *CYP1A2* (Table S2). These include non-human primates, rodents, even-toed ungulates and whales, other mammals, birds, fishes, other vertebrates, etc. In Ensembl release 84, *CYP1A2* has 69 orthologs from 60 species of chordates including 9 species of non-human primates, 8 species of rodents, 11 species of Laurasiatheria, 33 species of placental mammals, 7 species of Sauropsida, and 11 species of fishes (Figure 2 and Table S3). In GeneCards 4.1.1, *CYP1A2* has orthologs in 15 species including chimpanzee, dog, mouse, rat, etc. (Table S4).

The *CYP1B1* gene located on chromosome 2p22.2 contains 3 exons and 2 introns, encoding a 543-amino acid protein. CYP1B1 can metabolize some drugs and several endogenous substances, but its role points to the bioactivation of certain procarcinogens such as polycyclic aromatic hydrocarbons. *CYP1B1* is highly expressed in the nasal epithelium and lung, with a low expression in the liver, intestine, and kidney. In NCBI HomoloGene 68, *CYP1B1* has 11 homologenes in 11 species, including chimpanzee, Rhesus monkey, dog, cattle, mouse, rat, Xenopus, zebrafish, etc. Based on NCBI Annotation Pipeline, 156 organisms have orthologs with *CYP1B1* (Table S2). These include non-human primates, rodents, even-toed ungulates and whales, other mammals, birds, fishes, other vertebrates, etc. In Ensembl 84, *CYP1B1* has 64 orthologs from 61 species of chordates including 11 species of non-human primates, 8 species of rodents, 11 species of Laurasiatheria, 33 species of placental mammals, 7 species of Sauropsida, and 11 species of fishes (Figure 2 and Table S3). In GeneCards 4.1.1, *CYP1B1* has orthologs in 15 species including chimpanzee, cattle, dog, mouse, rat, etc. (Table S4).

CYP17A1 converts pregnenolone and progesterone to their corresponding 17α-hydroxylated metabolites and then to dehydroepiandrosterone (DHEA) and androstenedione. CYP17A1 maps to chromosome 10q24.3. CYP17A1 is conserved in chimpanzee, Rhesus monkey, dog, cow, mouse, rat, *Arabidopsis thaliana* and rice. In NCBI HomoloGene 68, CYP17A1 has 15 homologs in 8 species including chimpanzee, Rhesus monkey, dog, chicken, frog, zebrafish, etc. Based on NCBI Annotation Pipeline, 155 organisms have orthologs with CYP17A1 (Table S2). These include non-human primates, rodents, even-toed ungulates and whales, other mammals, birds, fishes, other vertebrates, etc. In Ensembl 84, *CYP17A1* has 129 orthologs from 62 species including 10 species of non-human primates, 8 species of rodents, 13 species of Laurasiatheria, 36 species of placental mammals, 6 species of Sauropsida, and 11 species of fishes (Figure 2 and Table S3). In GeneCards 4.1.1, *CYP17A1* has orthologs in 15 species including chimpanzee, cattle, dog, mouse, rat, opossum, etc. (Table S4).

CYP21A2 catalyzes steroid 21-hydroxylation, which is needed for the synthesis of mineralocorticoids and glucocorticoids in adrenal gland. *CYP21A2* contains 10 exons and 9 introns and displays relatively low sequence identity compared to other *CYP* members. *CYP21A2* resides a multiallelic, complex and tandem copy number variation of the major histocompatibility complex region on chromosome 6p21.3. *CYP21A2* is expressed primarily in the adrenal cortex but has a low level in brain and lymphocytes. Mutations in *CYP21A2* cause congenital adrenal hyperplasia. *CYP21A1P* is pseudogene also located on 6p21.3. *CYP21A2* is conserved in chimpanzee, Rhesus monkey, dog, cow, mouse, rat, chicken, zebrafish, and frog (Figure 2). In NCBI HomoloGene 68, *CYP21A2* has 9 homologs in 9 species including chimpanzee, Rhesus monkey, dog, mouse, rat, etc. Based on NCBI Annotation Pipeline, 106 organisms have orthologs with *CYP21A2* (Table S2). These include non-human primates, rodents, even-toed ungulates and whales, other mammals, birds, fishes, other vertebrates, etc. In Ensembl 84, *CYP21A2* has 120 orthologs from 53 species including 10 species of non-human primates, 7 species of rodents, 8 species of Laurasiatheria, 29 species of placental mammals, 6 species of Sauropsida, and 10 species of fish (Figure 2 and Table S3). In GeneCards 4.1.1, *CYP21A2* has orthologs in 11 species including chimpanzee, cattle, dog, mouse, rat, opossum, chicken, lizard, tropical clawed frog, zebrafish, and fruitfly (Table S4).

### 2.3. The Paralogs, Homologs and Orthologs of CYP2 Family

**Figure 3:**
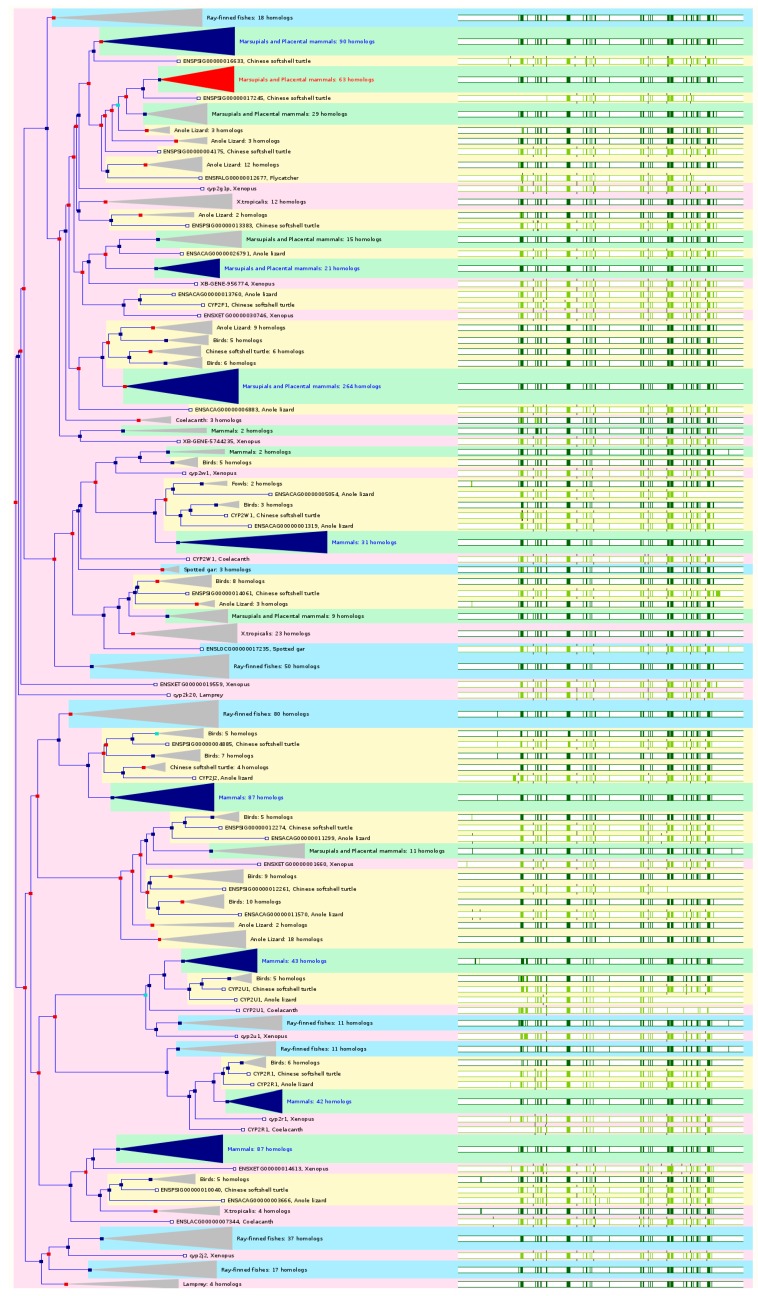

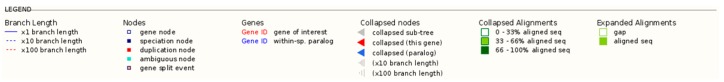
Gene tree for human *CYP2A6*, *2A7*, *2A13*, *2B6*, *2C8*, *2C9*, *2C19*, *2D6*, *2D7*, *2E1*, *2F1*, *2J2*, *2R1*, *2S1*, *2U1*, and *2W1* built using Ensembl 84. These *CYP2* family genes are paralogs to each other derived from the same ancestral gene via duplication events. The gene tree includes a total of 1254 genes from various species. The total number of speciation nodes is 741, and the number of duplication is 483. The number of ambiguous nodes is 29, and there is no gene split event.

The *CYP2ABFGST* cluster located on chromosome 19q13.2 includes *CYP2A*, *2B*, *2F*, *2G*, *2S*, and *2T* subfamilies. It contains six functional genes including *CYP2A6*, *2A7*, *2A13*, *2B6*, *2F1*, and *2S1* and seven pseudogenes including *CYP2A7P1*, *2B7P*, *2F2P*, *2G1P*, *2G2P*, *2T2P*, and *2T3P*. *CYP2G1P* carries a single nucleotide deletion in exon 2 and a 2.4-kb deletion between exons 3 and 7. *CYP2G2P* harbors two nonsense mutations in exons 1 and 3. The *CYP2ABFGST* cluster diverged through duplication events and inversions in the 80 Mya since the human and rodent lineages separated, resulting in 14 genes and 4 pseudogenes in rats, 12 active genes and 10 pseudogenes in mice, and 6 genes and 7 pseudogenes in humans. All the *CYP2* members are paralogs of each other (Figure 3 and Table 1).

CYP2A6 is a coumarin 7-hydroxylase that hydroxylates many drugs (e.g., tegafur, efavirenz, pilocarpine, and cyclophosphamide) and environmental and toxic compounds such as coumarin, nicotine, and nitrosamines. *CYP2A6* maps to chromosome 19q13.2 and consists of 9 exons. This gene is located within a 350-kb gene cluster on chromosome 19q13 together with *CYP2A7* and *2A13*, two *CYP2A7P* pseudogenes, and *CYP2B* and *CYP2F* subfamilies. CYP2A6 is mainly expressed in the liver. In NCBI HomoloGene 68, *CYP2A6* has 14 homologenes in 6 species including human *CYP2A7* and *2A13*, Rhesus monkey *CYP2A24*, mouse *Cyp2a4* and *2a5*, rat *Cyp2a3*, Xenopus *cyp2f2* and *2a6*, etc. Based on NCBI Annotation Pipeline, 3 species have orthologs with *CYP2A6* (Table S2). These include crab-eating macaque, Rhesus monkey, and western gorilla. In Ensembl 84, *CYP2A6* has 71 orthologs from 50 species of chordates including 7 species of non-human primates, 7 species of rodents, 12 species of Laurasiatheria, 29 species of placental mammals, 3 species of Sauropsida, and 11 species of fishes (Figure 3 and Table S3). In GeneCards 4.1.1, *CYP2A6* has orthologs in 9 species including chimpanzee, cattle, dog, mouse, rat, etc. (Table S4).

CYP2A7 is a hepatic enzyme, but its substrate specificity is unclear. The gene is located about 25 kb upstream of *CYP2A6*. It maps to chromosome 19q13.2 consisting of 9 exons. In NCBI HomoloGene 68, *CYP2A7* has 14 homologs in 6 species including human *CYP2A6* and *2A13*, Rhesus monkey *CYP2A24*, mouse *Cyp2a4* and *2a5*, rat *Cyp2a3*, etc. Based on NCBI Annotation Pipeline, 2 organisms have orthologs with *CYP2A7*, namely pig-tailed macaque and pygmy chimpanzee. In Ensembl 84, *CYP2A7* has 71 orthologs from 50 species of chordates including 7 species of non-human primates, 7 species of rodents, 12 species of Laurasiatheria, 29 species of placental mammals, 3 species of Sauropsida, and 11 species of fishes (Figure 3 and Table S3). In GeneCards 4.1.1, *CYP2A7* has orthologs in 9 species including chimpanzee, cattle, dog, mouse, rat, etc. (Table S4).

CYP2A13 is responsible for metabolic activation of hexamethylphosphoramide, *N*,*N*-dimethylaniline, and 4-(methylnitrosamino)-1-(3-pyridyl)-1-butanone, a tobacco-specific procarcinogen in the liver and other tissues. *CYP2A13* located in the *CYP2* cluster maps to chromosome 19q13.2. The nucleotide and protein sequences of CYP2A13 are highly similar to CYP2A6 with 93.5%–95.3% identity. *CYP2A13* is mainly expressed in the liver and also in nasal mucosa, lung, brain, and trachea. In NCBI HomoloGene 68, *CYP2A13* has 14 homologs in 6 species, including human *CYP2A6* and *2A7*, Rhesus monkey *CYP2A24*, mouse *Cyp2a4* and *2a5*, rat *Cyp2a3*, Xenopus *cyp2f2* and *2a6*, etc. Based on NCBI Annotation Pipeline, 3 species have orthologs with *CYP2A13*, namely Weddell seal, pygmy chimpanzee, and western gorilla (Table S2). In Ensembl 84, *CYP2A13* has 75 orthologs from 53 species of chordates including 10 species of non-human primates, 7 species of rodents, 12 species of Laurasiatheria, 32 species of placental mammals, 3 species of Sauropsida, and 11 species of fishes (Figure 3 and Table S3). In GeneCards 4.1.1, *CYP2A13* has orthologs in 12 species including chimpanzee, cattle, dog, mouse, rat, etc. (Table S4).

CYP2B6 metabolizes many drugs such as efavirenz, nevirapine, bupropion, and cyclophosphamide and activate certain procarcinogens and environmental compounds such as benzo(a)pyrene and some herbicides and pesticides. The gene maps to chromosome 19q13.2 together with *CYP2B7P*. It contains 9 exons encoding a 491-amino acid protein. *CYP2B6* is conserved in chimpanzee, Rhesus monkey, dog, cow, mouse, and rat. In NCBI HomoloGene 68, *CYP2B6* has 7 homologs in 7 species, including chimpanzee, Rhesus monkey, dog, cattle, mouse, and rat. Based on NCBI Annotation Pipeline, 25 organisms have orthologs with *CYP2B6* (Table S2). These include the *CYP2B6*-like or *CYP2B4* gene in non-human primates, pika, hedgehog, Arabian camel, degu, etc. In Ensembl 84, *CYP2B6* has 79 orthologs from 48 species of chordates including 8 species of non-human primates, 6 species of rodents, 11 species of Laurasiatheria, 29 species of placental mammals, 1 species of Sauropsida (Chinese softshell turtle), and 11 species of fishes (Figure 3 and Table S3). In GeneCards 4.1.1, *CYP2B6* has orthologs in 9 species including chimpanzee, cattle, dog, mouse, rat, etc. (Table S4).

CYP2F1 catalyzes the dehydrogenation of 3-methylindole, an endogenous toxin derived from the fermentation of tryptophan and bioactivates lung toxicants 4-ipomeanol, naphthalene, and styrene. This gene is mainly expressed in the lung, with very low or no expression in the liver, kidney, and intestine. *CYP2F1* was present in the common ancestor of chordates and is conserved in chimpanzee, Rhesus monkey, dog, mouse, and rat. In NCBI HomoloGene 68, *CYP2F1* has 5 homologs in 5 species, including chimpanzee, Rhesus monkey, dog, mouse, and rat. Based on NCBI Annotation Pipeline, 46 organisms have orthologs with *CYP2F1* (Table S2). These include non-human primates, rodents, other mammals, fishes, other vertebrates, etc. In Ensembl 84, *CYP2F1* has 45 orthologs from 36 species of chordates including 3 species of non-human primates, 5 species of rodents, 9 species of Laurasiatheria, 17 species of placental mammals, 1 species of Sauropsida, and 11 species of fishes (Figure 3 and Table S3). In GeneCards 4.1.1, *CYP2F1* has orthologs in 9 species including chimpanzee, cattle, dog, mouse, rat, etc. (Table S4).

Human CYP2S1 oxidizes a number of carcinogens via the peroxide shunt. CYP2S1 also metabolizes PGG_2_, PGH_2_, hydroperoxyeicosatetraenoic acids, and 13*S*-hydroperoxyoctadecadienoic acid, resulting in thromboxane A_2_ and other epoxyalcohols. The gene is mainly expressed in nasal epithelium, trachea, lung, stomach, small intestine, and spleen. *CYP2S1* is conserved in chimpanzee, dog, cow, mouse, and rat. In NCBI HomoloGene release 68, *CYP2S1* has 5 homologs in 5 species, including chimpanzee, dog, cattle, mouse, and rat. Based on NCBI Annotation Pipeline, 83 organisms have orthologs with *CYP2S1* (Table S2). These include non-human primates, rodents, even-toed ungulates and whales, other mammals, birds, fishes, other vertebrates, etc. In Ensembl 84, *CYP2S1* has 56 orthologs from 47 species of chordates including 9 species of non-human primates, 8 species of rodents, 4 species of Laurasiatheria, 28 species of placental mammals, 1 species of Sauropsida, and 11 species of fishes (Figure 3 and Table S3). In GeneCards 4.1.1, *CYP2S1* has orthologs in 10 species including chimpanzee, cattle, dog, mouse, rat, opossum, etc. (Table S4).

The human *CYP2C* genes located on chromosome 10q24 align with an order of Centriole–*2C18*–*2C19*–*2C9*–*2C8*–Telemere. The human *CYP2C* genes have a significant potential to recombine, since they contains many L1 LINE repetitive DNA sequences located primarily in intron 5. Both *CYP2C9* and *2C19* contain L1PA7, L1M4, L1MB5 and L1PA16 repeats in this intron. *CYP2C18* and *2C19* share L1PA5 repeats. Both *CYP2C8* and *2C19* carry an L1P repeat, but the two genes are on opposite strands. In mice, 14 of the 15 *Cyp2c* genes are located within a single cluster except for *Cyp2c44* which is 3.8 Mb away from the locus and has unique catalytic property, expression profile and regulation. In the current rat genome assembly Rnor_6.0, there are 13 *Cyp2c* genes including *2c6/2c37*, *2c7/2c39*, *2c11*, *2c12/2c40*, *2c13/2c38*, *2c22-2c24*, *2c26*, *2c29*, *2c66*, *2c79/2c65*, and *2c80/2c55*, which are located in a single cluster on rat chromosome 1q.

CYP2C8 metabolizes many drugs such as paclitaxel, amodiaquine, and methadone and several endogenous compounds such as AA and retinoid acid (RA). *CYP2C8* is conserved in chimpanzee, Rhesus monkey, mouse, and rat. In NCBI HomoloGene release 68, *CYP2C8* has 6 homologs in 4 species, including chimpanzee, Rhesus monkey, mouse, and rat. Based on NCBI Annotation Pipeline, 10 organisms have orthologs with *CYP2C8* (Table S2). These include chimpanzee, pygmy chimpanzee, Bolivian squirrel monkey, horse, etc. In Ensembl 84, *CYP2C8* has 163 orthologs from 59 species of chordates including 10 species of non-human primates, 7 species of rodents, 13 species of Laurasiatheria, 35 species of placental mammals, 7 species of Sauropsida, and 11 species of fishes (Figure 3 and Table S3). In GeneCards 4.1.1, *CYP2C8* has orthologs in 10 species including chimpanzee, cattle, dog, mouse, rat, opossum, etc. (Table S4).

CYP2C9 is abundant in the liver and metabolizes 15% of drugs that are metabolized by CYPs such as *S*-flurbiprofen, *S*-warfarin, tolbutamide, phenytoin, and diclofenac. *CYP2C9* is conserved in chimpanzee, Rhesus monkey, cow, mouse, and rat. In NCBI HomoloGene 68, *CYP2C9* has 8 homologs in 5 species, including chimpanzee, Rhesus monkey, cattle, mouse, rat, etc. Based on NCBI Annotation Pipeline, 7 organisms have orthologs with *CYP2C9*. These include chimpanzee, pygmy chimpanzee, Rhesus monkey, macaque, etc. In Ensembl 84, *CYP2C9* has 160 orthologs from 58 species of chordates including 8 species of non-human primates, 8 species of rodents, 13 species of Laurasiatheria, 34 species of placental mammals, 7 species of Sauropsida, and 11 species of fishes (Figure 3 and Table S3). In GeneCards 4.1.1, *CYP2C9* has orthologs in 7 species including chimpanzee, cattle, mouse, rat, etc. (Table S4).

CYP2C18 can metabolize several drugs including tolbutamide, phenytoin, and verapamil. The gene is mainly expressed in the liver, esophagus, stomach, and small intestine. The *CYP2C18* gene is conserved in Rhesus monkey, cow, mouse, rat, chicken, and mosquito. In NCBI HomoloGene 68, *CYP2C18* has 7 homologs in 6 species, including Rhesus monkey, cattle, mouse, rat, etc. Based on NCBI Annotation Pipeline, 27 organisms have orthologs with *CYP2C18* (Table S2). These include non-human primates, even-toed ungulates and whales, other mammals, etc. In Ensembl 84, *CYP2C18* has 123 orthologs from 53 species of chordates including 8 species of non-human primates, 5 species of rodents, 11 species of Laurasiatheria, 29 species of placental mammals, 7 species of Sauropsida, and 11 species of fishes (Figure 3 and Table S3). In GeneCards 4.1.1, *CYP2C18* has orthologs in 10 species including chimpanzee, dog, mouse, rat, etc. (Table S4).

CYP2C19 metabolizes about 10% of drugs that are metabolized by CYPs, such as phenytoin, omeprazole, and voriconazole. The gene is mainly expressed in the liver, small intestine, and gallbladder. *CYP2C19* is conserved in chimpanzee, Rhesus monkey, dog, cow, and rat. In NCBI HomoloGene 68, *CYP2C19* has 7 homologs in 6 species. These include chimpanzee, Rhesus monkey, dog, cow, rat, etc. Based on NCBI Annotation Pipeline, 7 organisms have orthologs with *CYP2C19* (Table S2). These include gray short-tailed opossum, pig, goat, etc. In Ensembl 84, *CYP2C19* has 157 orthologs from 55 species of chordates including 5 species of non-human primates, 8 species of rodents, 13 species of Laurasiatheria, 31 species of placental mammals, 7 species of Sauropsida, and 11 species of fishes (Figure 3 and Table S3). In GeneCards 4.1.1, *CYP2C19* has orthologs in 8 species including chimpanzee, cattle, dog, mouse, rat, etc. (Table S4).

*CYP2D6* belongs to a gene cluster consisting of highly homologous 2 functional genes and 1 pseudogene on chromosome 22q13. *CYP2D8P* encompasses multiple deletions and insertions in its exons. In Ensembl 84, this pseudogene produces one single transcript only that does not encode any functional proteins. The evolution of the human *CYP2D* locus results in inactivation of *CYP2D7* and *2D8P* and partial inactivation of *CYP2D6* (in ~10% Caucasian). Based on the identification and characterization of a non-functional *CYP2D7* gene and a *2D8P* pseudogene, gene duplication events may give rise to *CYP2D6* and *2D7*, and that gene conversion events occur later to generate *CYP2D8P*.

CYP2D6 accounts for only a small percentage of total hepatic CYPs (1.3%–4.3%), however, it metabolizes ~25% of the drugs that are metabolized by CYPs such as tamoxifen, imipramine, codeine, and dextromethorphan. *CYP2D6* is conserved in chimpanzee, Rhesus monkey, rat, chicken, and frog. In NCBI HomoloGene 68, *CYP2D6* has 8 homologs in 5 species. These include chimpanzee, Rhesus monkey, rat, chicken, and frog. Based on NCBI Annotation Pipeline, 77 organisms have orthologs with *CYP2D6* (Table S2). These include non-human primates, rodents, even-toed ungulates and whales, other mammals, birds, fishes, other vertebrates, etc. In Ensembl 84, *CYP2D6* has 97 orthologs from 49 species of chordates including 11 species of non-human primates, 8 species of rodents, 12 species of Laurasiatheria, 35 species of placental mammals, 7 species of Sauropsida, and 0 species of fishes (Figure 3 and Table S3). In GeneCards 4.1.1, *CYP2D6* has orthologs in 12 species including chimpanzee, mouse, rat, etc. (Table S4).

CYP2D7 is primarily expressed in brain cortex. In NCBI HomoloGene 68 and Orthologs from Annotation Pipeline, *CYP2D7* has no homolog and ortholog. In Ensembl 84, *CYP2D7* has 97 orthologs from 49 species of chordates including 11 species of non-human primates, 8 species of rodents, 12 species of Laurasiatheria, 35 species of placental mammals, 7 species of Sauropsida, and 0 species of fishes (Figure 3 and Table S3). In GeneCards 4.1.1, *CYP2D7* has only one ortholog in mouse (*Cyp2d37-ps*). The origin of the *CYP2D* subfamily could be traced back to before the divergence between amniotes and amphibians about 312 Mya. *CYP2D7* was derived from *CYP2D6* duplication in a stem lineage of humans and great apes. In fact, the origin of *CYP2D6* and *2D8P* in humans can be tracked back to a stem lineage of the New World monkeys and Catarrhini at the latest. Two functional CYP2Ds have been found in marmosets and macaques.

CYP2E1 metabolizes many low molecular weight xenobiotics such as acetaminophen, chlorzoxazone, halothane, and benzene. *CYP2E1* is located on chromosome 10q26.3 with 9 exons. CYP2E1 is mainly expressed in the liver. *CYP2E1* is conserved in chimpanzee, Rhesus monkey, dog, cow, mouse, and rat. In NCBI HomoloGene release 68, *CYP2E1* has 6 homologs in 6 species including chimpanzee, Rhesus monkey, dog, cattle, mouse, and rat. Based on NCBI Annotation Pipeline, 71 organisms have orthologs with *CYP2E1* (Table S2). These include non-human primates, rodents, even-toed ungulates and whales, other mammals, birds, fishes, other vertebrates, etc. In Ensembl 84, *CYP2E1* has 84 orthologs from 55 species of chordates including 9 species of non-human primates, 8 species of rodents, 12 species of Laurasiatheria, 32 species of placental mammals, 7 species of Sauropsida, and 11 species of fishes (Figure 3 and Table S3). In GeneCards 4.1.1, *CYP2E1* has orthologs in 6 species including chimpanzee, cattle, dog, mouse, rat, and opossum (Table S4).

CYP2J2 metabolizes AA, linoleic acid, and various drugs including certain antihistamine drugs (e.g., ebastine and terfenadine), mesoridazine, danazol, certain tyrosine kinase inhibitors (e.g., imatinib and sunitinib), fenbendazole, etc. In the human genome and GRCh38.p2, there is only one single *CYP2J2* gene, which maps to chromosome 1p31.3-p31.2. *CYP2J2* is highly expressed in heart, present at a lower level in the liver, intestine, brain, bladder, pancreas, placenta, and kidney. *CYP2J2* is conserved in chimpanzee, Rhesus monkey, dog, cattle, mouse, rat, chicken, zebrafish, frog, fruitfly, mosquito, and *C. elegans*. In NCBI HomoloGene 68, *CYP2J2* has as many as 51 homologs in 12 species, including chimpanzee, Rhesus monkey, dog, cattle, mouse, rat, etc. Based on NCBI Annotation Pipeline, 81 organisms have orthologs with *CYP2J2* (Table S2). These include non-human primates, rodents, even-toed ungulates and whales, other mammals, birds, fishes, other vertebrates, etc. In Ensembl 84, *CYP2J2* has 188 orthologs from 60 species of chordates including 11 species of non-human primates, 7 species of rodents, 14 species of Laurasiatheria, 37 species of placental mammals, 7 species of Sauropsida, and 11 species of fishes (Figure 3 and Table S3). In GeneCards 4.1.1, *CYP2J2* has orthologs in 15 species including chimpanzee, dog, mouse, rat, etc. (Table S4). Unlike humans, the mouse *Cyp2j* cluster has 7 functional genes including *Cyp2j5*, *2j6*, *2j8*, *2j9*, and *2j11-2j13* and 3 pseudogenes including *Cyp2j7-ps*, *2j14-ps*, and *2j15-ps*. This cluster has the unusual property that all the genes and pseudogene fragments are oriented in the same direction, which is not the case for other six *Cyp* clusters.

CYP2R1 is also known as vitamin D_3_ 25-hydroxylase that converts vitamin D into 25-hydroxyvitamin D_3_ (calcidiol). Calcidiol is subsequently converted by CYP27B1 (i.e., 25-hydroxyvitamin D_3_ 1-α-hydroxylase) to calcitriol, the active form of vitamin D_3_ that binds to vitamin D_3_ receptor which mediates most of the physiological actions of vitamin D_3_. *CYP2R1* maps to chromosome 11p15.2. CYP2R1 is mainly expressed in the liver and pancreas with the highest expression in the testes. *CYP2R1* is conserved from zebrafish and frog to human. In NCBI HomoloGene 68, *CYP2R1* has 9 homologs in 9 species including chimpanzee, Rhesus monkey, dog, cattle, mouse, rat, frog, etc. Based on NCBI Annotation Pipeline, 161 organisms have orthologs with *CYP2R1* (Table S2). These include non-human primates, rodents, even-toed ungulates and whales, other mammals, birds, fishes, other vertebrates, etc. In Ensembl 84, *CYP2R1* has 66 orthologs from 62 species of chordates including 10 species of non-human primates, 7 species of rodents, 14 species of Laurasiatheria, 37 species of placental mammals, 7 species of Sauropsida, and 11 species of fishes (Figure 3 and Table S3). In GeneCards 4.1.1, *CYP2J2* has orthologs in 11 species including chimpanzee, cattle, dog, mouse, etc. (Table S4).

CYP2U1 metabolizes AA, docosahexaenoic acid, other long-chain fatty acids, and endogenous *N*-arachidonoylserotonin. *CYP2U1* maps to chromosome 4q25. There is a high mRNA expression of *CYP2U1* in human thymus, with lesser expression in the heart, brain (mainly amygdala and prefrontal cortex), and platelets. The *CYP2U1* gene is conserved in chimpanzee, dog, cow, mouse, rat, chicken, zebrafish, frog, fruit fly, and *A. thaliana*. In NCBI HomoloGene 68, *CYP2U1* has 11 homologs in 10 species including chimpanzee, dog, cattle, mouse, rat, frog, etc. Based on NCBI Annotation Pipeline, 160 organisms have orthologs with *CYP2U1* (Table S2). These include non-human primates, rodents, even-toed ungulates and whales, other mammals, birds, fishes, other vertebrates, etc. In Ensembl 84, *CYP2U1* has 66 orthologs from 62 species of chordates including 11 species of non-human primates, 8 species of rodents, 13 species of Laurasiatheria, 37 species of placental mammals, 7 species of Sauropsida, and 11 species of fishes (Figure 3 and Table S3). In GeneCards 4.1.1, *CYP2U1* has orthologs in 13 species including chimpanzee, dog, mouse, rat, etc. (Table S4).

CYP2W1 catalyzes the oxidation of indole and certain lipids including lysolecithin and their stereoisomers and shows monooxygenase activity towards 3-methylindole and chlorzoxazone, but not AA. *CYP2W1* maps to chromosome 7p22.3. The gene contains 10 exons and encode a 490-amino acid protein. The 5-prime flanking region, first exon, and first intron of *CYP2W1* carry abundant CpG dinucleotides including 2 CpG islands. *CYP2W1* is mainly expressed in colorectal, hepatic and adrenal gland tumors, but it is rarely detected in normal tissues. *CYP2W1* is an ancient member of the *CYP* superfamily and it is conserved in chimpanzee, Rhesus monkey, dog, cow, mouse, rat, and chicken. In NCBI HomoloGene 68, *CYP2W1* has 7 homologs in 7 species including chimpanzee, Rhesus monkey, dog, cattle, mouse, rat, and chicken. Based on NCBI Annotation Pipeline, 108 organisms have orthologs with *CYP2W1* (Table S2). These include non-human primates, rodents, even-toed ungulates and whales, other mammals, birds, fishes, other vertebrates, etc. In Ensembl 84, *CYP2W1* has 93 orthologs from 52 species of chordates including 10 species of non-human primates, 7 species of rodents, 9 species of Laurasiatheria, 28 species of placental mammals, 7 species of Sauropsida, and 11 species of fishes (Figure 3 and Table S3). In GeneCards 4.1.1, *CYP2W1* has orthologs in 10 species including chimpanzee, cattle, dog, mouse, etc. (Table S4).

### 2.4. The Paralogs, Homologs and Orthologs of CYP3, 4, 5, and 46 Families

**Figure 4:**
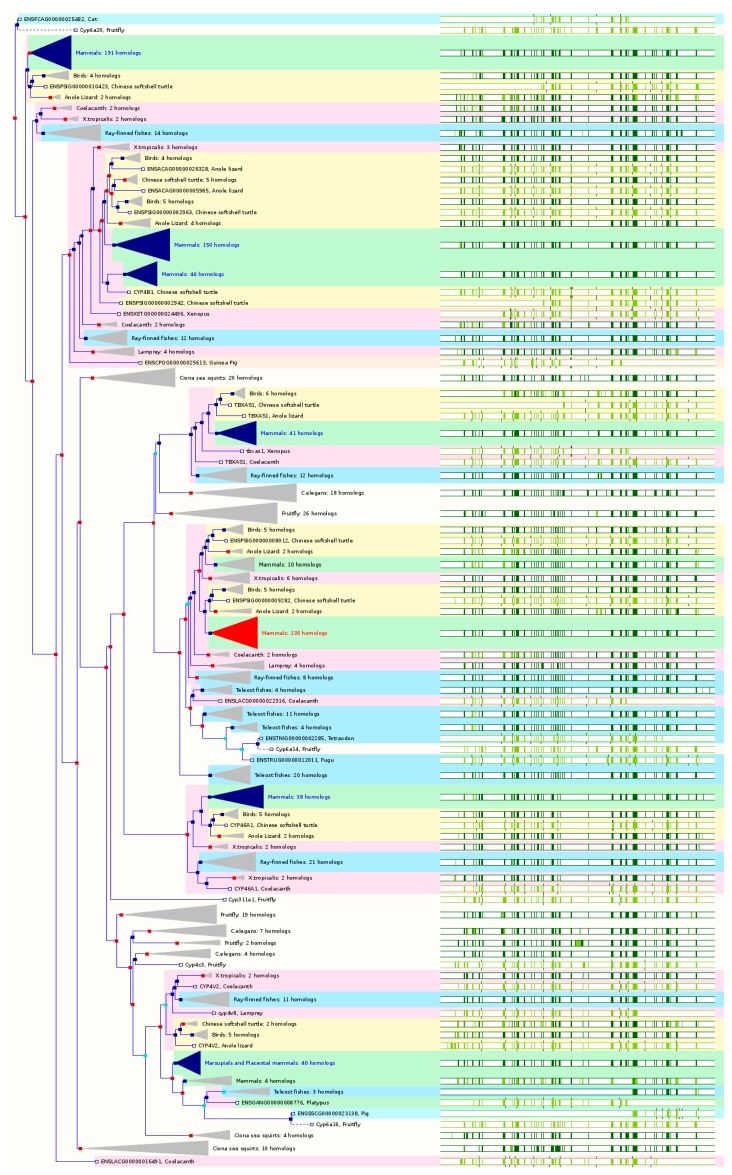

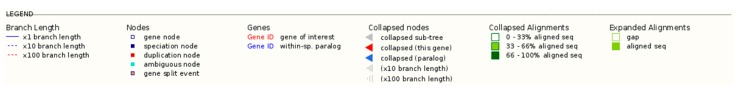
Gene tree for human *CYP3A4*, *3A5*, *3A7*, *3A43*, *4A11*, *4A22*, *4B1*, *4F2*, *4F3*, *4F8*, *4F11*, *4F12*, *4F22*, *4V2*, *4X1*, *4Z1*, *5A1/TBXAS1*, and *46A1* built using Ensembl 84. These *CYP3*, *4*, *5* and 46 family genes are paralogs to each other derived from the same ancestral gene via duplication events. The gene tree includes a total of 1008 genes from various species. The total number of speciation nodes is 558, and the number of duplication is 384. The number of ambiguous nodes is 31, and there are 4 gene split events.

The *CYP3A* gene cluster is located on chromosome 7q21.1 (Ensembl cytogenetic band: 7q22.1) and spans ~231 kb, containing 4 *CYP3A* genes: *CYP3A4*, *3A5*, *3A7* and *3A43*, as well as 2 pseudogenes including (*CYP3A51P/3A5P1* and *3A52P*/*3A5P2*). *CYP3A54P* and *3A137P* are two additional pseudogenes in *CYP3* family, which map to chromosome 7q22.1. The human CYP3A subfamily is involved in the oxidative metabolism of a wide range of substrates, including more than 50% of all currently marketed drugs, endogenous steroids and xenobiotics. *CYP3A4* and *3A5* are mainly expressed in the liver and intestine, while *CYP3A5* appears to be primarily expressed in extrahepatic tissues. *CYP3A4* is most abundantly expressed in the liver while *CYP3A5* expression at the protein level is only about 10.6% of that of CYP3A4. Both CYP3A4 and 3A5 share substrate specificity and so it is often difficult to identify their relative contribution to the overall metabolism of a substrate. *CYP3A4*, *3A5*, *3A7*, and *3A43* share paralogs from human *CYP* superfamily and orthologs from various species with slight differences only. CYP3A7 is a fetal-specific CYP. CYP3A43 has very low expression in the liver. In GeneCards 4.1.1, *CYP3*, *4*, and *5* members are paralogs to each other (Table 1). Ensembl 84 also includes *CYP46A1* as the paralog of *CYP3*, *4*, and *5* families (Figure 4 and Table 1).

*CYP3A4* is conserved in chimpanzee, Rhesus monkey, dog, cow, rat, chicken, frog, fruitfly, mosquito, *C. elegans*, and *M. oryzae*. In NCBI HomoloGene 68, *CYP3A4* has 27 homologs in 11 species, including chimpanzee, Rhesus monkey, dog, cattle, rat, etc. Based on NCBI Annotation Pipeline, 6 organisms have orthologs with *CYP3A4* (Table S2). These include chimpanzee, white-tufted-ear marmoset, drill, Bolivian squirrel monkey, etc. In Ensembl 84, *CYP3A4* has 148 orthologs from 60 species of chordates including 8 species of non-human primates, 8 species of rodents, 14 species of Laurasiatheria, 35 species of placental mammals, 7 species of Sauropsida, and 11 species of fishes (Figure 4 and Table S3). In GeneCards 4.1.1, *CYP3A4* has orthologs in 16 species including chimpanzee, cattle, dog, mouse, rat, etc. (Table S4).

*CYP3A5* is conserved in chimpanzee, Rhesus monkey, mouse, rat, and fruitfly. In NCBI HomoloGene 68, *CYP3A5* has 13 homologs in 5 species including chimpanzee, Rhesus monkey, mouse, rat, etc. Based on NCBI Annotation Pipeline, 10 organisms have orthologs with *CYP3A5* (Table S2). These include chimpanzee, Rhesus monkey, etc. In Ensembl 84, *CYP3A5* has 153 orthologs from 62 species of chordates including 10 species of non-human primates, 8 species of rodents, 14 species of Laurasiatheria, 35 species of placental mammals, 7 species of Sauropsida, and 11 species of fishes (Figure 4 and Table S3). In GeneCards 4.1.1, *CYP3A5* has orthologs in 13 species including chimpanzee, cattle, dog, mouse, rat, opossum, etc. (Table S4).

*CYP3A7* is conserved in chimpanzee, mouse, rat, chicken, zebrafish, frog, and rice. In NCBI HomoloGene 68, *CYP3A7* has 9 homologs in 7 species including chimpanzee, mouse, rat, chicken, Xenopus, etc. Based on NCBI Annotation Pipeline, 7 organisms have orthologs with *CYP3A7*, including chimpanzee, Rhesus monkey, macaque, and gorilla, etc. In Ensembl 84, *CYP3A7* has 150 orthologs from 61 species of chordates including 9 species of non-human primates, 8 species of rodents, 14 species of Laurasiatheria, 36 species of placental mammals, 7 species of Sauropsida, and 11 species of fishes (Figure 4 and Table S3). In GeneCards 4.1.1, *CYP3A7* has orthologs in 14 species including chimpanzee, cattle, dog, mouse, rat, opossum, *C. elegans*, etc. (Table S4). *CYP3A43* is conserved in primates only. In NCBI HomoloGene 68, *CYP3A43* has only 2 homologs in two primates including chimpanzee and Rhesus monkey. Based on NCBI Annotation Pipeline, 13 organisms have orthologs with *CYP3A43* (Table S2). These mainly include non-human primates and Ord’s kangaroo rat. In Ensembl 84, *CYP3A43* has 148 orthologs from 60 species of chordates including 8 species of non-human primates, 8 species of rodents, 14 species of Laurasiatheria, 35 species of placental mammals, 7 species of Sauropsida, and 11 species of fishes (Figure 4 and Table S3). In GeneCards 4.1.1, *CYP3A43* has orthologs in 12 species including chimpanzee, dog, mouse, zebrafish, fruitfly, *C. elegans*, etc. (Table S4).

There is a cluster of human *CYP4* genes on chromosome 1p33, including *CYP4A11*, *4A22*, *4B1*, *4A26P*, *4A27P*, *4A43P*, *4A44P*, *4X1*, *4Z1*, and *4Z2P*. The CYP4 proteins are the major fatty acid ω-hydorxylation which can hydroxylate the terminal ω-carbon and the ω-1 position of saturated and unsaturated fatty acids. They can also catalyze the ω-hydroxylation of various prostaglandins (PGs). *CYP4A11* is conserved in chimpanzee, mouse, and rat. In NCBI HomoloGene 68, *CYP4A11* has 5 homologs in 3 species including chimpanzee, mouse, and rat. Based on NCBI Annotation Pipeline, 26 organisms have orthologs with *CYP4A11* (Table S2). These mainly include non-human primates, rodents, even-toed ungulates and whales, other mammals, birds, fishes, other vertebrates, etc. In Ensembl 84, *CYP4A11* has 134 orthologs from 57 species of chordates including 10 species of non-human primates, 8 species of rodents, 11 species of Laurasiatheria, 32 species of placental mammals, 7 species of Sauropsida, and 11 species of fishes (Figure 4 and Table S3). In GeneCards 4.1.1, *CYP4A11* has orthologs in 13 species including chimpanzee, dog, mouse, rat, fruitfly, *C. elegans*, etc. (Table S4).

*CYP4A22* is conserved in chimpanzee, Rhesus monkey, dog, cow, mouse, rat, chicken, *A. thaliana*, and rice. In NCBI HomoloGene 68, *CYP4A22* has 13 homologs in 9 species, including chimpanzee, Rhesus monkey, dog, cow, mouse, rat, etc. Based on NCBI Annotation Pipeline, 3 organisms have orthologs with *CYP4A22*, including chimpanzee, pygmy chimpanzee, and Rhesus monkey (Table S2). In Ensembl 84, *CYP4A22* has 136 orthologs from 58 species of chordates including 11 species of non-human primates, 8 species of rodents, 11 species of Laurasiatheria, 33 species of placental mammals, 7 species of Sauropsida, and 11 species of fishes (Figure 4 and Table S3). In GeneCards 4.1.1, *CYP4A22* has orthologs in 11 species including chimpanzee, cattle, dog, mouse, rat, *A. thaliana*, etc. (Table S4).

*CYP4B1* is conserved in chimpanzee, Rhesus monkey, dog, cow, mouse, rat, chicken, zebrafish, and frog. In NCBI HomoloGene 68, *CYP4B1* has 12 homologs in 9 species, including chimpanzee, Rhesus monkey, dog, cattle, mouse, rat, chicken, zebrafish, etc. Based on NCBI Annotation Pipeline, 79 organisms have orthologs with *CYP4B1* (Table S2). These include non-human primates, rodents, even-toed ungulates and whales, other mammals, birds, fishes, other vertebrates, etc. In Ensembl 84, *CYP4B1* has 67 orthologs from 54 species of chordates including 10 species of non-human primates, 8 species of rodents, 12 species of Laurasiatheria, 35 species of placental mammals, 1 species of Sauropsida, and 11 species of fishes (Figure 4 and Table S3). In GeneCards 4.1.1, *CYP4B1* has orthologs in 13 species including chimpanzee, cattle, dog, mouse, rat, zebrafish, *C. elegans*, etc. (Table S4).

CYP4X1 is a so-called “orphan” enzyme, but it converts the natural endocannabinoid anandamide to a single monooxygenated product and also metabolizes AA. *CYP4X1* is mainly detected in the trachea, aorta, heart, liver, breast, brain, and prostate. *CYP4X1* is conserved in chimpanzee, Rhesus monkey, dog, cow, mouse, rat, *A. thaliana*, and rice. In NCBI HomoloGene 68, *CYP4X1* has 13 homologs in 8 species, including chimpanzee, dog, mouse, rat, etc. Based on NCBI Annotation Pipeline, 53 organisms have orthologs with *CYP4X1* (Table S2). These include non-human primates, rodents, even-toed ungulates and whales, other mammals, birds, fishes, other vertebrates, etc. In Ensembl 84, *CYP4X1* has 86 orthologs from 62 species of chordates including 10 species of non-human primates, 8 species of rodents, 14 species of Laurasiatheria, 37 species of placental mammals, 7 species of Sauropsida, and 11 species of fishes (Figure 4 and Table S3). In GeneCards 4.1.1, *CYP4X1* has orthologs in 10 species including chimpanzee, cattle, dog, mouse, rat, *A. thaliana*, etc. (Table S4).

CYP4Z1 is responsible for the in-chain hydroxylation of myristic acid and lauric acid. *CYP4Z1* is primarily expressed in mammary tissue. *CYP4Z1* is conserved in fish, mammal, and primate. In NCBI HomoloGene 68, *CYP4Z1* has only one homolog—chimpanzee *CYP4Z1*. Based on NCBI Annotation Pipeline, 14 organisms have orthologs with *CYP4Z1* (Table S2). These mainly include non-human primates, bottlenosed dolphin, etc. In Ensembl 84, *CYP4Z1* has 54 orthologs from 38 species of chordates including 5 species of non-human primates, 0 species of rodents, 0 species of Laurasiatheria, 5 species of placental mammals, 7 species of Sauropsida, and 11 species of fishes (Figure 4 and Table S3). In GeneCards 4.1.1, *CYP4Z1* has orthologs in 5 species including chimpanzee, mouse, opossum, platypus, and lizard (Table S4).

The human genome has 6 functional *CYP4F* genes including *CYP4F2*, *4F3*, *4F8*, *4F11*, *4F12*, and *4F22* and 5 pseudogenes including *4F9P*, *4F10P*, *4F23P*, *4F24P*, and *4F36P*, which are located at chromosome 19p13.12. The Entrez Gene cytogenetic band is chromosome 19p13.1. The CYP4F subfamily is able to metabolize several important endogenous eicosanoids such as AA, PGs, and leukotriene B_4_ (LTB_4_). These compounds can regulate many physiological functions such as inflammation and vasoconstriction. Both CYP4F3A and 4F3B catalyze LTB_4_ and AA ω-hydroxylation. CYP4Fs can convert AA to 20-HETE that regulates renal tubular and vascular functions. The ω-hydroxylated LTB_4_ is further metabolized to form 20-carboxy-LTB_4_, which can undergo B-oxidation from its ω-side and along with traditional β-oxidation from the C1 carbon, thus inactivating this pro-inflammatory agent. CYP4Fs appear to have a minor role in the biotransformation of therapeutic drugs. CYP4F2 can ω-hydroxylate vitamin E and vitamin K1 phytyl side chains, suggesting that this enzyme may play a role in the regulation of vitamin E status and synthesis of vitamin K-dependent clotting factors. CYP4F11 metabolizes erythromycin and ethylmorphine and CYP4F12 metabolizes ebastine with contribution from CYP2J2.

CYP4F2 is a major hepatic ω-hydroxylase that hydroxylates LTB_4_ and AA, inactivating LTB_4_ and forming the potent vasoconstrictor 20-hydroxyeicosatetraenoic acid (HETE), respectively. *CYP4F2* is part of a cluster of the *CYP4F* genes located on the band of chromosome 19p13.12. *CYP4F2* is conserved in chimpanzee, Rhesus monkey, cow, mouse, rat, and zebrafish. In NCBI HomoloGene 68, *CYP4F2* has 8 homologs, including chimpanzee, Rhesus monkey, cattle, mouse, rat, zebrafish, etc. Based on NCBI Annotation Pipeline, 5 organisms have orthologs with *CYP4F2* (Table S2). These include Rhesus monkey, yak, western gorilla, pygmy chimpanzee, and horse. In Ensembl 84, *CYP4F2* has 56 orthologs from 27 species of chordates including 6 species of non-human primates, 4 species of rodents, 8 species of Laurasiatheria, 18 species of placental mammals, 6 species of Sauropsida, and 0 species of fishes (Figure 4 and Table S3). In GeneCards 4.1.1, *CYP4F2* has orthologs in 12 species including chimpanzee, cattle, mouse, rat, zebrafish, fruitfly, *C. elegans*, etc. (Table S4).

CYP4F3 catalyzes the ω-hydroxylation of LTB_4_ and AA. The gene produces two tissue-specific splice variants, CYP4F3A (myeloid form) and CYP4F3B (liver form), but both transcripts are present in other tissues. *CYP4F3* is conserved in chimpanzee, Rhesus monkey, dog, cow, mouse, and rat. In NCBI HomoloGene 68, *CYP4F3* has 6 homologs in 6 species including chimpanzee, Rhesus monkey, cow, dog, mouse, and rat. Based on NCBI Annotation Pipeline, 15 organisms have orthologs with *CYP4F3* (Table S2). These mainly include non-human primates, rodents, even-toed ungulates and whales, other mammals, birds, fishes, other vertebrates, etc. In Ensembl 84, *CYP4F3* has 30 orthologs from 19 species of chordates including 2 species of non-human primates, 4 species of rodents, 3 species of Laurasiatheria, 10 species of placental mammals, 6 species of Sauropsida, and 0 species of fishes (Figure 4 and Table S3). In GeneCards 4.1.1, *CYP4F3* has orthologs in 12 species including chimpanzee, dog, mouse, rat, zebrafish, fruitfly, *C. elegans*, etc. (Table S4).

CYP4F8 catalyzes the ω-2 hydroxylation of AA and three stable PGH_2_ analogs but not PGD_2_, E_1_, E_2_, and F_2α_ and LTB_4_. *CYP4F8* is mainly expressed in epidermis, hair follicles, sweat glands, corneal epithelium, proximal renal tubules, and epithelial lining of the gut and urinary tract. *CYP4F8* is conserved in chimpanzee, Rhesus monkey, mouse, rat, and *A. thaliana*. In NCBI HomoloGene 68, *CYP4F8* has 12 homologs in 5 species including chimpanzee, Rhesus monkey, mouse, rat, etc. Based on NCBI Annotation Pipeline, 7 organisms have orthologs with *CYP4F8* (Table S2). These include Rhesus monkey, chimpanzee, green monkey, etc. In Ensembl 84, *CYP4F8* has 56 orthologs from 28 species of chordates including 7 species of non-human primates, 4 species of rodents, 8 species of Laurasiatheria, 19 species of placental mammals, 6 species of Sauropsida, and 0 species of fishes (Figure 4 and Table S3). In GeneCards 4.1.1, *CYP4F8* has orthologs in 6 species including chimpanzee, mouse, rat, fruitfly, *C. elegans*, and *A. thaliana* (Table S4).

CYP4F11 metabolizes LTB_4_ and AA but at a much lower activity than CYP4F2 and 4F3A/B. The *CYP4F11* gene is conserved in chimpanzee, mouse, rat, and chicken. In NCBI HomoloGene 68, *CYP4F11* has 4 homologs in 4 species including chimpanzee *CYP4F11*, mouse and rat *Cyp4f40*, and chicken *CYP4F22*. Based on NCBI Annotation Pipeline, 12 organisms have orthologs with *CYP4F11* (Table S2). These include chimpanzee *CYP4F11*, Sumatran orangutan, sooty mangabey, pygmy chimpanzee, etc. In Ensembl 84, *CYP4F11* has 58 orthologs from 28 species of chordates including 7 species of non-human primates, 4 species of rodents, 8 species of Laurasiatheria, 19 species of placental mammals, 6 species of Sauropsida, and 0 species of fishes (Figure 4 and Table S3). In GeneCards 4.1.1, *CYP4F11* has orthologs in 11 species including chimpanzee, cattle, mouse, rat, zebrafish, fruitfly, *C. elegans*, etc. (Table S4).

CYP4F12 catalyzes the hydroxylation of AA at C18 and C20, ω1 through ω3-hydroxylations of 9,11-diazo-PGH_2_ and 9,11-methanoepoxy-PGH_2_, and ω-oxidation of LTB_4_. In addition, CYP4F12 contributes to astemizole *O*-demethylation and hydroxylation of ebastine and terfenadine, with contributions from CYP3A4 and 2J2. *CYP4F12* is mainly expressed in the liver, kidney, colon, small intestine, urogenital tract, and heart. *CYP4F12* is conserved in chimpanzee, Rhesus monkey, mouse, rat, *A. thaliana*, and rice. In NCBI HomoloGene 68, *CYP4F12* has 7 homologs in 6 species including chimpanzee, Rhesus monkey, mouse, rat, etc. Based on NCBI Annotation Pipeline, 7 organisms have orthologs with *CYP4F12* (Table S2). These include chimpanzee, golden snub-nosed monkey, western gorilla, olive baboon, pygmy chimpanzee, etc. In Ensembl 84, *CYP4F12* has 58 orthologs from 28 species of chordates including 7 species of non-human primates, 4 species of rodents, 8 species of Laurasiatheria, 19 species of placental mammals, 6 species of Sauropsida, and 0 species of fishes (Figure 4 and Table S3). In GeneCards 4.1.1, *CYP4F12* has orthologs in 14 species including chimpanzee, dog, mouse, rat, zebrafish, fruitfly, *C. elegans*, etc. (Table S4).

CYP4F22 catalyzes the ω-3 hydroxylation of 20:4*n*-6 and synthesizes the ω-hydroxy fatty acids in the ceramide. *CYP4F22* is conserved in chimpanzee, Rhesus monkey, dog, cow, mouse, rat, frog, *A. thaliana*, and rice. In NCBI HomoloGene 68, *CYP4F22* has 9 homologs in 9 species including chimpanzee, Rhesus monkey, dog, cow, mouse, rat, frog, etc. Based on NCBI Annotation Pipeline, 92 organisms have orthologs with *CYP4F22* (Table S2). These include non-human primates, rodents, even-toed ungulates and whales, other mammals, birds, fishes, other vertebrates, etc. In Ensembl 84, *CYP4F22* has 44 orthologs from 43 species of chordates including 9 species of non-human primates, 6 species of rodents, 13 species of Laurasiatheria, 33 species of placental mammals, 6 species of Sauropsida, and 0 species of fishes (Figure 4 and Table S3). In GeneCards 4.1.1, *CYP4F22* has orthologs in 15 species including chimpanzee, dog, mouse, rat, zebrafish, *A. thaliana*, etc. (Table S4).

CYP4V2 is a selective ω-hydroxylase of saturated, medium-chain fatty acids with relatively high catalytic efficiency toward myristic acid and also hydroxylates the ω-3 polyunsaturated fatty acids such as docosahexaenoic acid and eicosapentaenoic acid. *CYP4V2* is an unusual *CYP4* member in that it resides on chromosome 4q35.2, separate from the *CYP4ABXZ* and *CYP4F* clusters on chromosomes 1 and 19. The mRNA of *CYP4V2* is found in the heart, brain, placenta, lung, liver, skeletal muscle, kidney, pancreas, retina, retinal pigment epithelium, and lymphocytes. The protein has very low sequence identity (31%–37%) to other CYP4 members. *CYP4V2* is conserved in chimpanzee, Rhesus monkey, dog, cow, mouse, rat, chicken, fruitfly, mosquito, frog, and *C. elegans*. In NCBI HomoloGene 68, *CYP4V2* has 12 homologs in 11 species including chimpanzee, Rhesus monkey, dog, cow, mouse, rat, frog, etc. Based on NCBI Annotation Pipeline, 153 organisms have orthologs with *CYP4V2* (Table S2). These include non-human primates, rodents, even-toed ungulates and whales, other mammals, birds, fishes, other vertebrates, etc. In Ensembl 84, *CYP4V2* has 76 orthologs from 61 species of chordates including 10 non-human primates, 8 species of rodents, 13 species of Laurasiatheria, 34 species of placental mammals, 7 species of Sauropsida, and 10 species of fishes (Figure 4 and Table S3). In GeneCards 4.1.1, *CYP4V2* has orthologs in 16 species including chimpanzee, dog, mouse, rat, zebrafish, fruitfly, *C. elegans*, etc. (Table S4).

TBXAS1 catalyzes the conversion of PGH_2_ to thromboxane, which causes vasoconstriction and platelet aggregation. *TBXAS1* maps to chromosome 7q34-q35. TBXAS1 is expressed in platelets, erythroleukemia cells, human monocytes, leukocytes, and kidney interstitial dendritic reticulum cells, with a low expression in the liver and lung. *TBXAS1* is conserved in dog, cow, mouse, rat, chicken, zebrafish, fruitfly, *A. thaliana*, rice, and frog. In NCBI HomoloGene 68, *TBXAS1* has 18 homologs in 10 species including dog, cattle, mouse, rat, chicken, Xenopus, zebrafish etc. Based on NCBI Annotation Pipeline, 154 organisms have orthologs with *TBXAS1* (Table S2). These include non-human primates, rodents, even-toed ungulates and whales, other mammals, birds, fishes, other vertebrates, etc. In Ensembl 84, *TBXAS1* has 106 orthologous genes from 61 species of chordates including 11 species of non-human primates, 8 species of rodents, 12 species of Laurasiatheria, 35 species of placental mammals, 7 species of Sauropsida, and 11 species of fishes (Figure 4 and Table S3). In GeneCards 4.1.1, *TBXAS1* has orthologs in 16 species including chimpanzee, cattle, dog, mouse, zebrafish, fruitfly, *C. elegans*, etc. (Table S4).

### 2.5. The Paralogs, Homologs and Orthologs of CYP7, 8, and 39 Families

**Figure 5:**
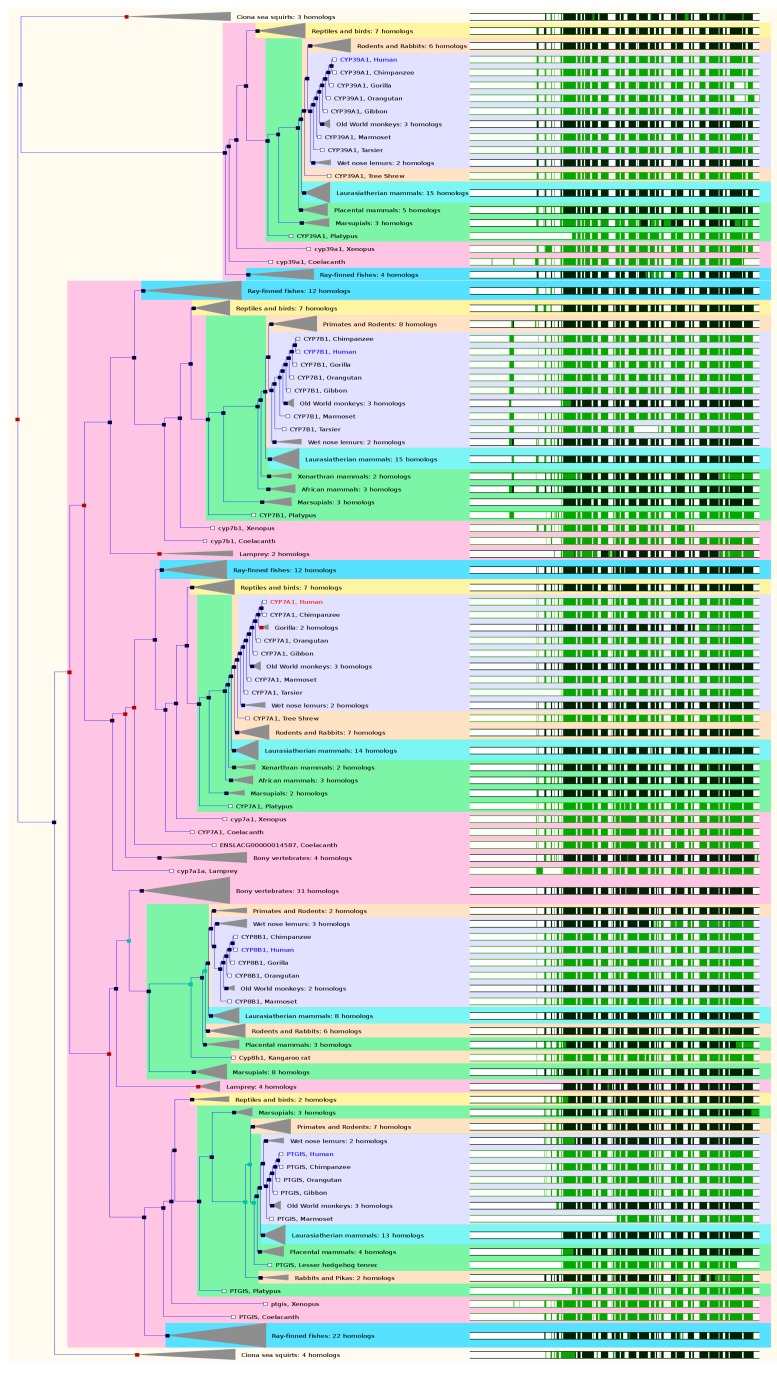

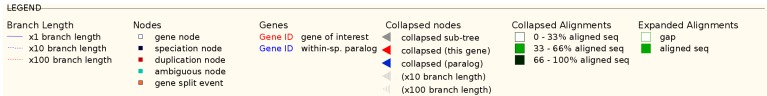
Gene tree for human *CYP7A1*, *7B1*, *8A1/PTGIS*, *8B1*, and *39A1* built using Ensembl 84. These *CYP7*, *8*, and *39* family genes are paralogs to each other derived from the same ancestral gene via duplication events. The gene tree includes a total of 340 genes from various species. The total number of speciation nodes is 287, and the number of duplication is 41. The number of ambiguous nodes is 10, and there is only 1 gene split event.

CYP7B1 shares 40% amino acid sequence identity with CYP7A1. Human *CYP7A1* and *7B1* share identical paralogs and orthologs. CYP7A1 (called cholesterol 7α-hydroxylase) catalyzes the first and major rate-limiting step in the classical, neutral pathway for bile acid biosynthesis. The *CYP7A1* gene maps to chromosome 8q11-q12 and its promoter region contains recognition sequences for a number of liver-specific transcription factors. The gene spans 10,059 bases with 7 exons, encoding a 504-amino acid enzyme. In both GeneCards 4.1.1 and Ensembl 84, *CYP7A1* has 4 paralogs: *CYP7B1*, *PTGIS*, *8B1*, and *39A1* (Figure 5 and Table 1).

*CYP7A1* is conserved in chimpanzee, Rhesus monkey, dog, cow, mouse, rat, chicken, zebrafish, and frog. In NCBI HomoloGene 68, *CYP7A1* has 9 homologs in 9 species including chimpanzee, Rhesus monkey, dog, cow, mouse, rat, chicken, Xenopus, and zebrafish. Based on NCBI Annotation Pipeline, 163 organisms have orthologs with *CYP7A1* (Table S2). These include non-human primates, rodents, even-toed ungulates and whales, other mammals, birds, fishes, other vertebrates, etc. In Ensembl 84, *CYP7A1* has 68 orthologs from 64 species including 11 species of non-human primates, 8 species of rodents, 14 species of Laurasiatheria, 38 species of placental mammals, 7 species of Sauropsida, and 11 species of fishes (Figure 5 and Table S3). In GeneCards 4.1.1, *CYP7A1* has orthologs in 12 species including chimpanzee, cattle, dog, mouse, rat, zebrafish, etc. (Table S4).

CYP7B1 is involved in metabolism of oxysterols, sex hormones and neurosteroids. The gene spans 211,029 bases with 6 exons, encoding a 506-amino acid enzyme. CYP7B1 shares 40% amino acid sequence identity with CYP7A1. *CYP7B1* is conserved in chimpanzee, Rhesus monkey, dog, cow, mouse, rat, chicken, zebrafish, and frog. In NCBI HomoloGene 68, *CYP7B1* has 9 homologs in 9 species including chimpanzee, Rhesus monkey, dog, mouse, rat, etc. Based on NCBI Annotation Pipeline, 150 organisms have orthologs with *CYP7B1* (Table S2). These include non-human primates, rodents, even-toed ungulates and whales, other mammals, birds, fishes, other vertebrates, etc. In Ensembl 84, *CYP7A1* has 70 orthologs from 65 species including 11 species of non-human primates, 8 species of rodents, 14 species of Laurasiatheria, 38 species of placental mammals, 7 species of Sauropsida, and 11 species of fishes (Figure 5 and Table S3). In GeneCards 4.1.1, *CYP7B1* has orthologs in 12 species including chimpanzee, cattle, dog, mouse, rat, zebrafish, etc. (Table S4).PTGIS (prostaglandin I_2_ synthase) catalyzes the conversion of PGH_2_ to prostacyclin, a potent vasodilator and inhibitor of platelet aggregation. *PTGIS* maps to chromosome 20q13.13. *PTGIS* is conserved in chimpanzee, Rhesus monkey, dog, cow, mouse, rat, zebrafish, and frog. In NCBI HomoloGene 68, *PTGIS* has 8 homologs in 8 species including chimpanzee, Rhesus monkey, dog, cattle, mouse, rat, frog, and zebrafish. Based on NCBI Annotation Pipeline, 100 organisms have orthologs with *PTGIS*/*CYP8A1* (Table S2). These include non-human primates, rodents, even-toed ungulates and whales, other mammals, birds, fishes, other vertebrates, etc. In Ensembl 84, *PTGIS* has 70 orthologs from 56 species including 9 species of non-human primates, 8 species of rodents, 13 species of Laurasiatheria, 35 species of placental mammals, 2 species of Sauropsida (Anole lizard and Chinese softshell turtle), and 11 species of fishes (Figure 5 and Table S3). In GeneCards 4.1.1, human *PTGIS* has orthologs in 11 species including chimpanzee, cattle, dog, mouse, rat, tropical clawed frog, zebrafish, etc. (Table S4).

CYP8B1 is involved in bile acid synthesis and is responsible for the conversion of 7α-hydroxy-4-cholesten-3-one into 7α,12α-dihydroxy-4-cholesten-3-one. *CYP8B1* maps to chromosome 3p22.1 and it contains only one single exon and lacks introns. *CYP8B1* is conserved in chimpanzee, Rhesus monkey, dog, mouse, rat, chicken, zebrafish, and frog. In NCBI HomoloGene 68, *CYP8B1* has 11 homologs in 8 species including chimpanzee, Rhesus monkey, dog, mouse, rat, etc. Based on NCBI Annotation Pipeline, 120 organisms have orthologs with *CYP8B1* (Table S2). These include non-human primates, rodents, even-toed ungulates and whales, other mammals, birds, fishes, other vertebrates, etc. In Ensembl 84, *CYP8B1* has 71 orthologs from 50 species including 8 species of non-human primates, 8 species of rodents, 8 species of Laurasiatheria, 27 species of placental mammals, 6 species of Sauropsida, 10 species of fishes (Figure 5 and Table S3). In GeneCards 4.1.1, *CYP8B1* has orthologs in 13 species including chimpanzee, cattle, dog, mouse, rat, opossum, zebrafish, etc. (Table S4).

CYP39A1 catalyzes 7α-hydroxylation of oxysterols including 25-hydroxycholesterol, 27-hydroxycholesterol and 24-hydroxycholesterol, while CYP7B1 is called oxysterol 7α-hydroxylase 1. *CYP39A1* maps to chromosome 6p12.3, spans 103,079 bases, and contains 14 exons. *CYP39A1* is conserved in chimpanzee, Rhesus monkey, dog, cow, mouse, rat, chicken, zebrafish, *M. oryzae*, and frog. In NCBI HomoloGene 68, *CYP39A1* has 10 homologs in 10 species including chimpanzee, Rhesus monkey, dog, mouse, rat, frog, etc. Based on NCBI Annotation Pipeline, 145 organisms have orthologs with *CYP39A1* (Table S2). These include non-human primates, rodents, even-toed ungulates and whales, other mammals, birds, fishes, other vertebrates, etc. In Ensembl 84, *CYP39A1* has 58 orthologs from 55 species including 10 species of non-human primates, 7 species of rodents, 14 species of Laurasiatheria, 37 species of placental mammals, 7 species of Sauropsida (birds and reptiles), and 3 species of fishes (Figure 5 and Table S3). In GeneCards 4.1.1, *CYP39A1* has orthologs in 13 species including chimpanzee, dog, mouse, rat, zebrafish, etc. (Table S4).

### 2.6. The Paralogs, Homologs and Orthologs of CYP11, 19, 24, 27, and 46 Families

The CYP11 members are important enzymes that participate in steroid biosynthesis and metabolism. The production of glucocorticoids and mineralocorticoids occurs in the adrenal gland and the final steps are catalyzed by three mitochondrial CYPs, namely CYP11A1, 11B1, and 11B2. *CYP11B1* shows close homology to the *CYP11B2* gene, which encodes aldosterone synthase and is normally expressed only in the zona glomerulosa. Both *CYP11B* genes map to chromosome 8q21, while *CYP11A1* is located on chromosome 15q24.1. All CYP11 members are mitochondrial enzymes. *CYP19A1*, *24A1*, *CYP27* family, and *46A1* are the paralogs of *CYP11* family members.

**Figure 6:**
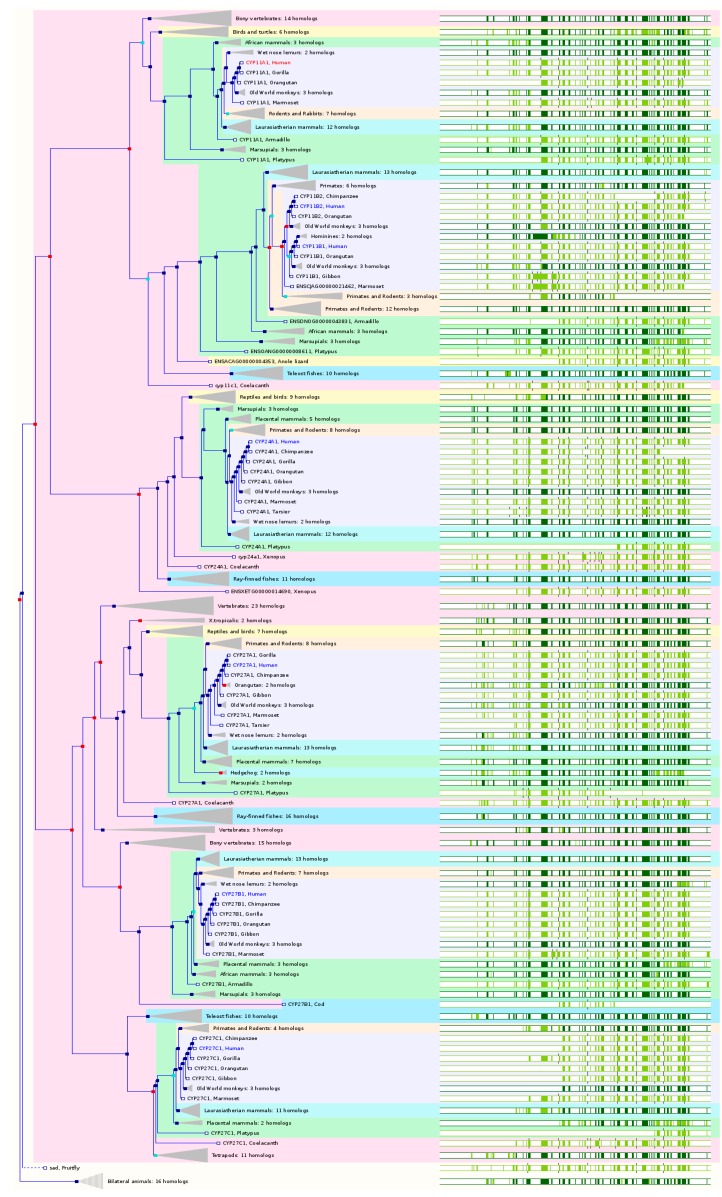

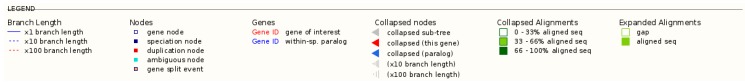
Gene tree for human *CYP11A1*, *11B1*, *11C1*, *24A1*, *27A1*, *27B1*, and *27C1* built using Ensembl 84. These *CYP11*, *24*, and *27* family genes are paralogs to each other derived from the same ancestral gene via duplication events. The gene tree includes a total of 410 genes from various species. The total number of speciation nodes is 344, and the number of duplication is 52. The number of ambiguous nodes is 13, and there is no gene split event.

CYP11A1 catalyzes the conversion of cholesterol to pregnenolone, which is the first and rate-limiting step in the synthesis of the steroid hormones. *CYP11A1* maps to chromosome 15q23-q24. *CYP11A1* is conserved in chimpanzee, dog, cow, mouse, rat, chicken, zebrafish, and frog. In NCBI HomoloGene 68, *CYP11A1* has 9 homologs in 8 species including chimpanzee, dog, cow, mouse, rat, etc. Based on NCBI Annotation Pipeline, 148 organisms have orthologs with *CYP11A1*. These include non-human primates, rodents, even-toed ungulates and whales, other mammals, birds, fishes, other vertebrates, etc. In Ensembl 84, *CYP11A1* has 61 orthologs from 58 species including 8 species of non-human primates, 7 species of rodents, 12 species of Laurasiatheria, 31 species of placental mammals, 6 species of Sauropsida, 11 species of fishes (Figure 6 and Table S3). In GeneCards 4.1.1, *CYP11A1* has orthologs in 14 species including chimpanzee, dog, mouse, rat, zebrafish, fruitfly, *C. elegans*, etc. (Table S4).

CYP11B1 catalyzes the 11β-hydroxylation of deoxycortisol to form cortisol and also catalyzes 18 or 19-hydroxylation of steroids and the aromatization of androstendione to estrone. *CYP11B1* maps to chromosome 8q21. The gene spans 7,491 bases with 11 exons and 10 introns. *CYP11B1* is conserved in chimpanzee, Rhesus monkey, mouse, rat, zebrafish, and frog. In NCBI HomoloGene release 68, *CYP11B1* has 6 homologs: chimpanzee, Rhesus monkey, mouse, rat, frog, and zebrafish. Based on NCBI Annotation Pipeline, 23 organisms have orthologs with *CYP11B1*. These include chimpanzee, macaque, Rhesus monkey, green monkey, etc. In Ensembl 84, *CYP11B1* has 64 orthologs from 52 species including 11 species of non-human primates, 8 species of rodents, 9 species of Laurasiatheria, 32 species of placental mammals, 1 species of Sauropsida (Anole lizard), and 10 species of fishes (Figure 6 and Table S3). In GeneCards 4.1.1, *CYP11B1* has orthologs in 13 species including chimpanzee, dog, mouse, rat, zebrafish, fruitfly, *C. elegans*, etc. (Table S4).

CYP11B2 catalyzes the conversion of 11-deoxycorticosterone to aldosterone via corticosterone and 18-hydroxycorticosterone. The gene spans 7285 bases with 10 exons and 9 introns, encoding a 503-amino acid enzyme. CYP11B2 is expressed in the adrenal cortex. *CYP11B2* is conserved in Rhesus monkey, dog, cow, mouse, and rat. In NCBI HomoloGene 68, *CYP11B2* has 6 homologs in 5 species including Rhesus monkey, dog, cattle, mouse, and rat. Based on NCBI Annotation Pipeline, 13 organisms have orthologs with *CYP11B2* (Table S2). These include Rhesus monkey, crab-eating macaque, pygmy chimpanzee, domestic cat, cattle, etc. In Ensembl 84, *CYP11B2* has 62 orthologs from 50 species including 9 species of non-human primates, 8 species of rodents, 9 species of Laurasiatheria, 30 species of placental mammals, 1 species of Sauropsida (Anole lizard), and 10 species of fishes (Figure 6 and Table S3). In GeneCards 4.1.1, *CYP11B2* has orthologs in 12 species including chimpanzee, dog, mouse, rat, zebrafish, fruitfly, *C. elegans*, etc. (Table S4).

CYP19A1 catalyzes the formation of aromatic C18 estrogens from C19 androgens. *CYP19A1* maps to chromosome 15q21.1 and contains 13 exons. CYP19A1 is expressed in the ovaries, testes, placenta, fetal liver, adipose tissue, bone, vasculature smooth muscle, and brain. *CYP19A1* is conserved in chimpanzee, Rhesus monkey, dog, cow, mouse, rat, chicken, zebrafish, and frog. In NCBI HomoloGene 68, *CYP19A1* has 10 homologs in 9 species including chimpanzee, Rhesus monkey, dog, cattle, mouse, rat, etc. Based on NCBI Annotation Pipeline, 155 organisms have orthologs with *CYP19A1* (Table S2). These include non-human primates, rodents, even-toed ungulates and whales, other mammals, birds, fishes, other vertebrates, etc. In Ensembl 84, *CYP19A1* has 75 orthologs from 62 species including 11 species of non-human primates, 8 species of rodents, 14 species of Laurasiatheria, 38 species of placental mammals, 7 species of Sauropsida, and 11 species of fishes (Table S3). In GeneCards 4.1.1, *CYP19A1* has orthologs in 14 species including chimpanzee, dog, mouse, rat, zebrafish, fruitfly, etc. (Table S4).

CYP24A1 is called vitamin D_3_ 24-hydroxylase that catalyzes the 24-hydroxylation of calcidiol (25-hydroxyvitamin D_3_) and calcitriol (1α,25-dihydroxyvitamin D_3_). This gene maps to chromosome 20q13.2 and contains 12 exons. The enzyme is present in both proximal and distal kidney tubules. *CYP24A1* is conserved in chimpanzee, Rhesus monkey, dog, cow, mouse, rat, chicken, zebrafish, frog, fruitfly, mosquito, and *C. elegans*. In NCBI HomoloGene 68, *CYP24A1* has 15 homologs in 12 species, including chimpanzee, Rhesus monkey, dog, cattle, mouse, rat, frog, zebrafish, etc. Based on NCBI Annotation Pipeline, 163 organisms have orthologs with *CYP24A1* (Table S2). These include non-human primates, rodents, even-toed ungulates and whales, other mammals, birds, fishes, other vertebrates, etc. In Ensembl 84, *CYP24A1* has 68 orthologs from 64 species including 11 species of non-human primates, 8 species of rodents, 12 species of Laurasiatheria, 36 species of placental mammals, 7 species of Sauropsida, and 11 species of fishes (Figure 6 and Table S3). In GeneCards 4.1.1, *CYP24A1* has orthologs in 14 species including chimpanzee, cattle, dog, mouse, rat, fruitfly, *C. elegans*, etc. (Table S4).

The human *CYP27* family contains three functional members: *CYP27A1*, *27B1*, and *27C1*. Both sterol 27-hydroxylase and 25-hydroxy-D_3_ 1α-hydroxylase are assigned to the CYP27 family since they share >40% sequence identity, while sterol 27-hydroxylase is assigned to the A subfamily and 25-hydroxy-D_3_ 1α-hydroxylase to the B subfamily of CYP27 since their protein sequences are <55% identical. The paralogs of *CYP27* family include *CYP11* family, *19A1*, *24A1*, and *46A1* (Figure 6). CYP27A1 is called vitamin D_3_ 25-hydroxylase and sterol 27-hydroxylase catalyzing the first step in the oxidation of the side chain of sterol intermediates. *CYP27A1* maps to chromosome 2q35 and contains 9 exons. Mutations in *CYP27A1* cause cerebrotendinous xanthomatosis, a rare autosomal recessive lipid storage disease. *CYP27A1* is conserved in chimpanzee, Rhesus monkey, dog, cow, mouse, rat, chicken, zebrafish, frog, fruit fly, and mosquito. In NCBI HomoloGene 68, *CYP27A1* has 16 homologs in 11 species including chimpanzee, Rhesus monkey, dog, cattle, mouse, rat, zebrafish etc. Based on NCBI Annotation Pipeline, 139 organisms have orthologs with *CYP27A1* (Table S2). These include non-human primates, rodents, even-toed ungulates and whales, other mammals, birds, fishes, other vertebrates, etc. In Ensembl 84, *CYP27A1* has 78 orthologs from 65 species including 11 species of non-human primates, 8 species of rodents, 13 species of Laurasiatheria, 37 species of placental mammals, 7 species of Sauropsida, and 11 species of fishes (Figure 6 and Table S3). In GeneCards 4.1.1, *CYP27A1* has orthologs in 14 species including chimpanzee, cattle, dog, mouse, rat, zebrafish, fruitfly, etc. (Table S4).

CYP27B1 is called 25-hydroxyvitamin D_3_ 1α-hydroxylase that hydroxylates 25-hydroxyvitamin D_3_ at the 1α position, resulting in 1α,25-dihydroxyvitamin D_3_, the active form of vitamin D_3_. *CYP27B1* maps to chromosome 12q12-q14 and has 9 exons. Mutations in this gene causes vitamin D-dependent rickets, type I and hypocalcemic vitamin D-dependent rickets. *CYP27B1* is conserved in in chimpanzee, Rhesus monkey, dog, cow, mouse, rat, zebrafish, and frog. In NCBI HomoloGene 68, *CYP27B1* has 9 homologs in 9 species including chimpanzee, Rhesus monkey, dog, cattle, mouse, rat, frog, etc. Based on NCBI Annotation Pipeline, 116 organisms have orthologs with *CYP27B1* (Table S2). These include non-human primates, rodents, even-toed ungulates and whales, other mammals, birds, fishes, other vertebrates, etc. In Ensembl 84, *CYP27B1* has 61 orthologs from 58 species including 10 species of non-human primates, 8 species of rodents, 14 species of Laurasiatheria, 36 species of placental mammals, 2 species of Sauropsida, and 11 species of fishes (Figure 6 and Table S3). In GeneCards 4.1.1, *CYP27B1* has orthologs in 12 species including chimpanzee, dog, mouse, rat, zebrafish, fruitfly, *C. elegans*, etc. (Table S4).

*CYP27C1* maps to chromosome 2q14.3 and contains 14 exons. The gene spans 36,243 bases and encodes a 372-amino acid protein. *CYP27C1* is conserved in chimpanzee, Rhesus monkey, dog, cow, chicken, zebrafish, frog, and *M. oryzae*. In NCBI HomoloGene 68, *CYP27C1* has 11 homologs in 10 species including chimpanzee, Rhesus monkey, dog, mouse, rat, zebrafish, etc. Based on NCBI Annotation Pipeline, 132 organisms have orthologs with *CYP27C1* (Table S2). These include non-human primates, rodents, even-toed ungulates and whales, other mammals, birds, fishes, other vertebrates, etc. In Ensembl 84, *CYP27C1* has 53 orthologs from 51 species including 9 species of non-human primates, 4 species of rodents, 11 species of Laurasiatheria, 25 species of placental mammals, 7 species of Sauropsida, and 10 species of fishes (Figure 6 and Table S3). In GeneCards 4.1.1, *CYP27C1* has orthologs in 11 species including chimpanzee, cattle, dog, zebrafish, fruitfly, etc. (Table S4).

CYP46A1 (called cholesterol 24-hydroxylase) converts cholesterol to 24*S*-hydroxycholesterol in the brain. Cholesterol cannot pass the blood-brain barrier, but 24*S*-hydroxycholesterol can be secreted in the brain into the circulation to be returned to the liver for catabolism. *CYP46A1* maps to chromosome 14q32.1. *CYP46A1* is conserved in chimpanzee, Rhesus monkey, dog, cow, mouse, rat, chicken, frog, *M. oryzae*, *A. thaliana*, and rice. In NCBI HomoloGene 68, *CYP46A1* has 15 homologs in 12 species including chimpanzee, Rhesus monkey, dog, mouse, rat, frog, zebrafish, etc. Based on NCBI Annotation Pipeline, 148 organisms have orthologs with *CYP46A1* (Table S2). These include non-human primates, rodents, even-toed ungulates and whales, other mammals, birds, fishes, other vertebrates, etc. In Ensembl 84, *CYP46A1* has 69 orthologs from 57 species including 11 species of non-human primates, 7 species of rodents, 12 species of Laurasiatheria, 34 species of placental mammals, 7 species of Sauropsida, and 11 species of fishes (Figure 4 and Table S3). In GeneCards 4.1.1, *CYP46A1* has orthologs in 15 species including chimpanzee, cattle, dog, mouse, rat, zebrafish, fruitfly, *A. thaliana*, etc. (Table S4).

### 2.7. The Paralogs, Homologs and Orthologs of CYP26 and 51 Families

**Figure 7:**
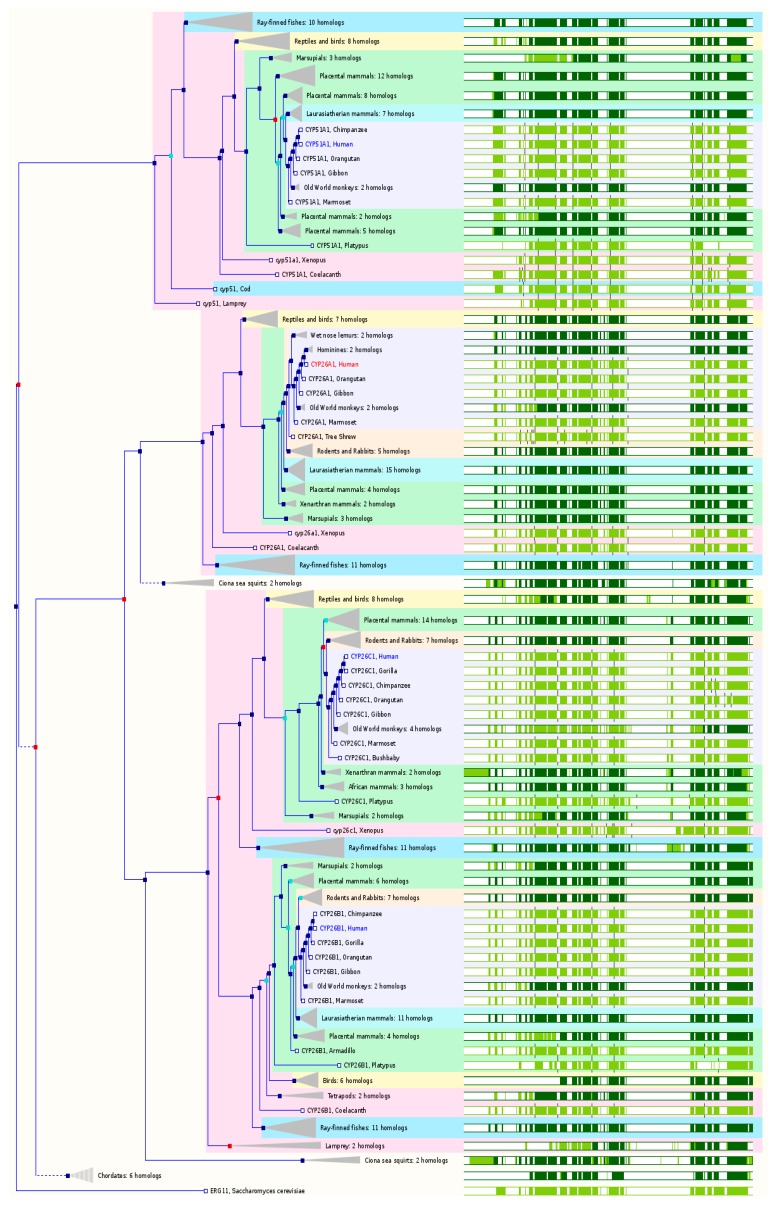

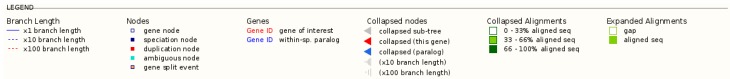
Gene tree for human *CYP26A1*, *26B1*, *26C1*, and *51A1* built using Ensembl 84. These *CYP26* and *CYP51* family genes are paralogs to each other derived from the same ancestral gene via duplication events. The gene tree includes a total of 260 genes from various species. The total number of speciation nodes is 232, and the number of duplication is 12. The number of ambiguous nodes is 15, and there is no gene split event.

In the human genome, there are three members of in the *CYP26* family: *26A1*, *26B1*, and *26C1*. These three members are all RA hydroxylases with similar substrate specificity but different tissue-specific expression patterns. CYP26A1 is called retinoic acid 4-hydroxylase with both 4-hydroxylation and 18-hydroxylation activities, acting on all-*trans*-RA and its stereoisomer 9-*cis*-RA. *CYP26A1* maps to chromosome 10q23-q24 and has 8 exons. In both GeneCards 4.1.1 and Ensembl 84, *CYP26* members are the paralogs of *CYP51A1* (Figure 7 and Table 1).

CYP26A1 has been detected in different cell lines with different tissue origins including kidney, liver, breast, intestine, and lung. Mutations in *CYP26A1* causes keratomalacia and caudal regression syndrome. *CYP26A1* is conserved in the chimpanzee, Rhesus monkey, dog, cow, mouse, rat, chicken, zebrafish, frog, *A. thaliana*, and rice. In NCBI HomoloGene 68, *CYP26A1* has 19 homologs in 11 species including chimpanzee, Rhesus monkey, dog, mouse, rat, frog, zebrafish etc. Based on NCBI Annotation Pipeline, 156 organisms have orthologs with *CYP26A1* (Table S2). These include non-human primates, rodents, even-toed ungulates and whales, other mammals, birds, fishes, other vertebrates, etc. In Ensembl 84, *CYP26A1* has 62 orthologs from 61 species including 9 species of non-human primates, 8 species of rodents, 14 species of Laurasiatheria, 35 species of placental mammals, 7 species of Sauropsida, and 11 species of fishes (Figure 7 and Table S3). In GeneCards 4.1.1, *CYP26A1* has orthologs in 16 species including chimpanzee, dog, mouse, rat, African clawed frog, zebrafish, fruitfly, baker’s yeast, *A. thaliana*, etc. (Table S4).

CYP26B1 is a critical regulator of all-*trans*-RA levels by the specific inactivation of all-*trans*-RA to hydroxylated forms. This gene maps to chromosome 2p13.2 and contains 8 exons. The gene spans 18,801 bases with 6 exons and encodes a 512-amino acid protein. Mutations in this gene are associated with radiohumeral fusions and other skeletal and craniofacial anomalies and lethal occipital encephalocele-skeletal dysplasia syndrome. *CYP26B1* is conserved in chimpanzee, Rhesus monkey, dog, cow, mouse, rat, zebrafish, frog, and *A. thaliana*. In NCBI HomoloGene 68, *CYP26B1* has 19 homologs in 10 species including chimpanzee, Rhesus monkey, dog, mouse, rat, frog, zebrafish, etc. Based on NCBI Annotation Pipeline, 153 organisms have orthologs with *CYP26B1* (Table S2). These include non-human primates, rodents, even-toed ungulates and whales, other mammals, birds, fishes, other vertebrates, etc. In Ensembl 84, *CYP26B1* has 64 orthologs from 62 species including 11 species of non-human primates, 8 species of rodents, 13 species of Laurasiatheria, 36 species of placental mammals, 6 species of Sauropsida, and 11 species of fishes (Figure 7 and Table S3). In GeneCards 4.1.1, *CYP26B1* has orthologs in 13 species including chimpanzee, cattle, dog, mouse, rat, tropical clawed frog, zebrafish, fruitfly, baker’s yeast, *A. thaliana*, etc. (Table S4).CYP26C1 is involved in the catabolism of all-*trans*- and 9-*cis*-RA, and thus contributes to the regulation of RA levels in cells and tissues. Like *CYP26A1*, this gene maps to chromosome 10q23.33. The gene spans 7,434 bases with 6 exons and encodes a 522-amino acid protein Mutations of *CYP26C1* causes focal facial dermal dysplasia 4 and focal facial dermal dysplasia. *CYP26C1* is the conserved in chimpanzee, cow, mouse, rat, chicken, zebrafish, frog, and *A. thaliana*. In NCBI HomoloGene 68, *CYP26C1* has 8 homologs in 8 species including chimpanzee, cattle, mouse, rat, chicken, frog, zebrafish, etc. Based on NCBI Annotation Pipeline, 139 organisms have orthologs with *CYP26C1* (Table S2). These include non-human primates, rodents, even-toed ungulates and whales, other mammals, birds, fishes, other vertebrates, etc. In Ensembl 84, *CYP26C1* has 63 orthologs from 60 species including 10 species of non-human primates, 8 species of rodents, 11 species of Laurasiatheria, 34 species of placental mammals, 7 species of Sauropsida, and 11 species of fishes (Figure 7 and Table S3). In GeneCards 4.1.1, *CYP26C1* has orthologs in 14 species including chimpanzee, dog, mouse, rat, zebrafish, fruitfly, *A. thaliana*, etc. (Table S4).

CYP51A1 is called lanosterol 14α-demethylase/sterol 14α-demethylase which are found in yeast, plants, fungi, animals and even prokaryotes, suggesting this is among the oldest of the *CYP* genes. CYP51A1 is a common target of antifungal drugs (e.g., miconazole and ketoconazole), which inhibit CYP51A1 activity and formation of ergosterol. This gene has 11 exons and maps to chromosome 7q21.2. *CYP51A1* is conserved in chimpanzee, Rhesus monkey, dog, cow, mouse, rat, chicken, zebrafish, frog, *Saccharomyces cerevisiae*, *Kluyveromyces lactis*, *Eremothecium gossypii*, *Schizosaccharomyces pombe*, *M. oryzae*, *A. thaliana*, and rice. In NCBI HomoloGene 68, *CYP51A1* has 16 homologs in 16 species including chimpanzee, Rhesus monkey, dog, cattle, mouse, rat, frog, zebrafish, etc. Based on NCBI Annotation Pipeline, 162 organisms have orthologs with *CYP51A1* (Table S2). These include non-human primates, rodents, even-toed ungulates and whales, other mammals, birds, fishes, other vertebrates, etc. In Ensembl 84, *CYP51A1* has 64 orthologs from 63 species including 10 species of non-human primates, 8 species of rodents, 14 species of Laurasiatheria, 37 species of placental mammals, 7 species of Sauropsida, and 11 species of fishes (Figure 7 and Table S3). In GeneCards 4.1.1, *CYP51A1* has orthologs in 19 species including chimpanzee, cattle, dog, mouse, rat, zebrafish, baker’s yeast, etc. (Table S4).

### 2.8. The Paralogs and Homologs of CYP20A1

*CYP20A1* maps to chromosome 2q33.2 and contains 14 exons. The gene spans 67,400 bases, encoding a 462-amino acid protein. This protein lacks one amino acid of the conserved heme binding site and also lacks the conserved I-helix motif AGX(D,E)T, suggesting that its substrate may carry its own oxygen. As an “orphan” CYP, the substrate specificity, structure, function and regulation of CYP20A1 are still unknown. In both GeneCards 4.1.1 and Ensembl 84, there is no paralog for *CYP20A1*. *CYP20A1* is conserved in chimpanzee, Rhesus monkey, dog, cow, mouse, rat, chicken, zebrafish, and frog. In NCBI HomoloGene 68, *CYP20A1* has 9 homologs in 9 species, including chimpanzee, Rhesus monkey, dog, mouse, rat, frog, and zebrafish. Based on NCBI Annotation Pipeline, 160 organisms have orthologs with *CYP20A1* (Table S2). These include non-human primates, rodents, even-toed ungulates and whales, other mammals, birds, fishes, other vertebrates, etc. In Ensembl 84, *CYP20A1* has 66 orthologs from 62 species including 11 species of non-human primates, 7 species of rodents, 14 species of Laurasiatheria, 37 species of placental mammals, 7 species of Sauropsida, and 10 species of fishes (Table S3). In GeneCards 4.1.1, *CYP20A1* has orthologs in 14 species including chimpanzee, dog, mouse, rat, zebrafish, etc. (Table S4).

## 3. Discussion

After 3.5 billion years of evolution, the number of species on the earth has expanded considerably. Each genome consists of a unique gene inventory, which determines the specific phenotype and interactions with the environment. Genotypic and phenotypic diversity have been observed in all species at the protein, DNA, and organismal levels, and this diversity is correlated with environmental variation and stress. The time frame for the evolution of the genus Homo out of the chimpanzee–human last common ancestor is roughly 10 to 2 Mya, that of *Homo sapiens* out of *Homo erectus* roughly 1.8 to 0.2 Mya. According to genetic and fossil evidence, archaic *Homo sapiens* evolved to anatomically modern humans solely in Africa, between 200,000 and 100,000 years ago, with members of one branch leaving Africa by 60,000 years ago and over time replacing earlier human populations such as Neanderthals and *Homo erectus*. Humans, and presumably most vertebrates, have genes not found in invertebrate animals like Drosophila and *C. elegans*. These include genes encoding antibodies and T cell receptors for antigen, the transplantation antigens of the major histocompatibility complex, cell-signaling molecules including the many types of cytokines, the molecules that participate in blood clotting, and mediators of apoptosis.

The human genome includes 57 protein-coding *CYP* genes, which play a key role in the biotransformation of a large number of xenobiotics such as drugs and environmental compounds and physiologically important endogenous compounds. Most human *CYP* genes are scattered widely across their genomes, but there are some exceptions. Human *CYP* genes often occur in clusters, with several related genes, pseudogenes and detritus exons aligned in tandem [10]. Mouse and human each have 30 *CYP* genes that lie outside the seven gene clusters. These CYP genes are distributed on all chromosomes except chromosomes 5, 16, and 17. Five clusters of closely related genes are located on chromosomes 1, 7, and 10 (one cluster each) and chromosome 19 (two clusters). Clusters of human *CYP* genes are found at different chromosome regions including 1p31 (*CYP2J*), 1p33 (*CYP4ABXZ* cluster), 7q22 (*CYP3A* cluster), 10q24 (*CYP2C* cluster), 19p13 (*CYP4F cluster*), 19q13 (*CYP2ABFGST* cluster), and 22q13 (*CYP2D* cluster). In each of these clusters, the *CYP* genes are adjacent, with no other confirmed genes interspersed among them. Within each cluster, all the genes encode closely related enzymes with many cases of apparent gene duplication since the split from the rodent lineage [46,47]. Almost all xenobiotic-metabolizing CYP enzymes belonging to CYP1-4 families are located in these gene clusters. Each of the human *CYP* clusters had a syntenic counterpart cluster in mice. Very few of the genes in these clusters could be assigned as one-to-one orthologs due to continuing gene duplication and deletion events on both lineages. These syntenic gene clusters must have originated from a shared ancestral gene or genes, with gene duplications and losses resulting in lineage-specific groups of related genes. Genomic clustering of structurally and functionally related genes such as *CYPs* are also found in other species.

It is postulated that approximately one and a half billion years ago, the first of the gene expansions gave rise to the families of *CYPs* that are primarily involved in the metabolism of endogenous fatty acids and cholesterol (e.g., CYP4 and 11 families). Around 900 Mya, another expansion of the gene family is speculated to have resulted in several of the endogenous steroid-synthesizing CYP families (e.g., CYP19, 21 and 27 families). A dramatic expansion of several CYP families, including those known or suspected of being involved in xenobiotic metabolism (e.g., CYP2, 3, 4 and 6), commenced about 400 Mya. Phylogenetic analyses of CYPs suggest that they are also among the most rapidly evolving of genes which is a characteristic that is needed to protect the cells from the injuries when exposed to increasing toxic xenobiotic compounds [3,36,37].

It is generally assumed that orthologs have the same biological functions in different species, and duplication events produce paralogs that evolve new functions [39,40]. Clear delineation of orthologous relationships between *CYP* genes is obviously indispensable for the reconstruction of the evolution of species and their genomes in the post-genomic era. To achieve this objective, we have systematically studied the relationships of 57 human *CYPs* with those from other species. A sequence alignment and phylogenetic study have clearly shown the evolution of human *CYPs* from one ancestral gene and the key features as a functional group of heme-containing oxidative enzymes. The structural motifs identified include “AGXDTT”, “EXXR”, and “CXG”. Several residues including Glu242, Arg245, Phe310, and Cys316 are found to be well conserved in all human CYPs. In particular, Cys316 plays a central role in heme-binding where iron acts as a source/sink of electrons for reduction/oxidation reactions.

We have applied two approaches to identify the paralogs of human *CYPs*: GeneCards and Ensembl. Both methods produce similar results with slight differences (Table 2). Both GeneCards and Ensembl have identified *CYP3*, *4*, and *5* members are paralogs to each other, but Ensembl predicts that *CYP46A1* shares the ancestor with these genes. GeneCards predicts that *CYP46A1* shares the ancestor with *CYP11*, *24*, and *27* members. Both GeneCards and Ensembl have found that *CYP7* and *8* members are the paralogs of *CYP39A1*. GeneCards predicts that *CYP11*, *24*, *27* and *46* members are the paralogs of *CYP19A1*, but Ensembl has told us that *CYP19A1* has no paralogs at all. Both GeneCards and Ensembl predict that *CYP26* members are the paralogs of *CYP51A1*. The differences in the predicted paralogs of human *CYPs* may reflect the differences in the algorithms and cutoff values for inclusion and exclusion used by the two approaches.

Many important genes are conserved across species despite billions of years of intervening evolution and exposure to dramatically changed environment. The wide application of comparative genomics is essential in order to map knowledge across different species. Sequences of genes that share a common ancestry are typically refined into orthologs, which are pairs of genes that started diverging via speciation event, and paralogues, which are pairs of genes that started diverging via gene duplication [39,40]. Many approaches and databases have been developed to identify orthologs. We have adopted six approaches/databases to predict the orthologs of human *CYPs*, including NCBI, Ensembl Compara, GeneCards, OMA, PANTHER, and TreeFam. Since the species sets used by these six databases are different, it is not surprising to see a very different number of species and orthologs of a human *CYP* gene. However, the predicted orthologs of human *CYPs* based on these approaches are comparable. For example, they all predict that *CYP46A1* and *51A1* have orthologs in non-human primate, rodent, placental mammal, fish, frog, rice, rice blast fungus, and *A. thaliana*, suggesting these two enzymes play an essential role in maintaining the cellular functions and biotransformation of key endogenous compounds across animal, yeast, and plant. CYP46A1 is a cholesterol 24-hydroxylase while CYP51A1 is a lanosterol 14α-demethylase [48]. Other CYPs that are significantly involved in the metabolism of important endogenous compounds are also well conserved across species based on our data. CYP4 members are major fatty acid ω-hydroxylases [49]. These enzymes remove excess free fatty acids to prevent lipotoxicity, catabolize leukotrienes and prostanoids including prostaglandins, thromboxanes and prostacylins, and result in bioactive metabolites from arachidonic acid ω-hydroxylation. CYP7A1 and 7B1 are 7α-hydroxylases of steroids. CYP11A1, 11B1, and 11B2 are involved in steroid biosynthesis. CYP17A1 is present in adrenal cortex and has steroid 17α-hydroxylase and 17,20-lyase activities for steroids. CYP19A1 is an aromatase present in gonads, brain, and adipose tissue that catalyzes aromatization of androgens to estrogens. CYP21A2 is detected in adrenal cortex and has 21-hydroxylase activity toward steroids [50,51]. CYP26A1, 26B1, and 26C1 are retinoid acid hydroxylases. Moreover, CYP39A1 catalyzes 7α-hydroxylation of 24-hydroxycholesterol. All these enzymes are conserved in various species and indicate their key role in the survival of these species. CYP51 is involved in cholesterol biosynthesis, whereas CYP 7A1, 27A1, 46A1, 7B1, 39A1, and 8B1 are the key enzymes in cholesterol catabolism to bile acids, the major route of cholesterol elimination [52]. Conversion of cholesterol to steroids are initiated by CYP11A1, and CYP3A4 contributes to bile acid biosynthesis as well [52]. Six CYPs including CYP11 family and three type II CYPs including CYP17A1, 19A1 and 21A2 play indispensable roles in the biosynthesis of steroids. The key CYP enzymes in the bile acid biosynthetic pathways are CYP7A1, 8B1, 27A1 and 7B1. Biosynthesis and metabolism of cholesterol, bile acids and oxysterols involve CYP3A4, 7A1, 7B1, 8B1, 27A1, 39A1, 46A1, and 51A1. CYPs have many physiologically relevant functions including regulation of vascular tone in the cardiovascular system, ion transport in the kidney, inflammation and immune system, the secretion of pancreatic peptide hormones, cell proliferation and programmed cell death [8,26,53,54,55]. CYPs participate in cellular functions such as the metabolism of eicosanoids, the biosynthesis of cholesterol and bile acids, synthesis and metabolism of steroids and vitamin D_3_, synthesis and degradation of biogenic amines, and the hydroxylation of RA and presumably other morphogens [49,56,57]. The metabolites of these endogenous compounds often have important physiological activities that regulate cellular metabolism, death and survival.

Clusters of *CYP* genes are often present in the genomes of various species. The processes of sequential tandem gene duplication events can lead to large clusters of *CYP* genes on chromosomes, and these are often striking landmarks of the CYPomes in various species. Clusters of related *CYP* families are called “clans”. There are 10 *CYP* clans in humans: clans 2, 3, 4, 7, 19, 20, 26, 46, and 51, and the mitochondrial clan [47]. CYP families within a single clan have likely been diverged from a common ancestor gene. Clusters of human *CYP* genes are found at different chromosome regions including 1p31 (*CYP2J*), 1p33 (*CYP4ABXZ* cluster), 7q22 (*CYP3A* cluster), 10q24 (*CYP2C* cluster), 19p13 (*CYP4F cluster*), 19q13 (*CYP2ABFGST* cluster), and 22q13 (*CYP2D* cluster). In each of these clusters, the *CYP* genes are adjacent, with no other confirmed genes interspersed among them. Within each cluster, all the genes encode closely related enzymes with many cases of apparent gene duplication since the split from the rodent lineage [47]. Almost all xenobiotic-metabolizing CYP enzymes belonging to CYP1-4 families are located in these gene clusters. Each of the human *CYP* clusters had a syntenic counterpart cluster in mice. Very few of the genes in these clusters could be assigned as one-to-one orthologs due to continuing gene duplication and deletion events on both lineages. These syntenic gene clusters must have originated from a shared ancestral gene or genes, with gene duplications and losses resulting in lineage-specific groups of related genes. Genomic clustering of structurally and functionally related genes such as *CYPs* are also found in the other species.

The *CYP1A2* gene may arise via duplication of *CYP1A1* about 350 Mya during the evolution of mammals and birds based on phylogenetic analysis of *CYP1A* genes. The human *CYP2* family has 4 clusters: *CYP2ABFGST*, *CYP2C*, *CYP2D*, and *CYP2J*. The *CYP2ABFGST* cluster diverged through duplication events and inversions in the 80 Mya since the human and rodent lineages separated, resulting in 14 genes and 4 pseudogenes in rats, 12 active genes and 10 pseudogenes in mice, and 6 genes and 7 pseudogenes in humans. Both CYP1 and CYP2 families belong to the CYP2 clan [58]. The CYP1 family is considered to diverge from the CYP2 family more than 420 Mya. The CYP1 family has four subfamilies (1A, 1B, 1C, and 1D), and these subfamilies diverge in the ancestor of vertebrates. Fish and amphibians express all these 4 subfamily members. The CYP1A and 1B subfamilies are conserved from fish to humans, whereas primates lack CYP1D, and mammals lack CYP1C [33]. Birds have two *CYP1A* genes, *CYP1A4* and *1A5*, which are orthologous to mammalian *CYP1A1* and *1A2* [59]. CYP1C members are found in several bird genomes, but not in quail [60]. The *CYP1C* genomic region is highly conserved among vertebrates. *CYP1B* and *1C* genes derive from duplication of a common ancestor gene. Tissue distribution of *CYP1B* and *1C* transcripts in birds resembles that found in zebrafish, suggesting that these genes have similar functions in diverse vertebrates [60]. The CYP1A and 1B subfamilies are conserved from the fish to human, whereas primates lack CYP1D, and mammals lack CYP1C [61,62].

The CYP2 family, comprising at least 42 subfamilies (2A–2H, 2J–2N, 2P–2Z, 2AA–2AH, 2AJ–2AK, 2AM, 2AN, and 2AP–2AU), is the most dominant in Clan 2. The CYP2 family is considered to arise from a single ancestral vertebrate *CYP2* gene. CYP2B, 2E and 2S subfamilies are specific to mammals, while the CYP2A/G and 2F subfamilies are present only in mammals and reptiles [63]. About half of the CYP2 subfamilies are non-mammalians: 2H derives from chicken; 2K, 2M, 2N and 2P are from fish; 2L is from lobster; 2Q, 2AC, 2AM, 2AN, 2AP, 2AQ, 2AR, 2AS, and 2AT from Xenopus; 2AA and 2AD from fish; 2AG, 2AH, 2AJ, and 2AK from green anole lizard; and 2AU from oyster. The first five (2A–2E) are present in mammalian liver with differing levels, while CYP2F members are selectively expressed in lung tissues, and have been implicated as important catalysts in the formation of reactive intermediates from several pneumotoxic chemicals. Avian CYP2Hs are orthologous to human CYP2C62P, rat Cyp2c23, and mouse Cyp2c44 [64]. CYP2R and 2U are present in all vertebrates. CYP2B, 2E and 2S are specific to mammals, while the 2A, 2G and 2F subfamilies are present only in mammals and reptiles. These five subfamilies (except the CYP2E subfamily) diverged successively to result in the *CYP2* cluster in an ancestor of mammals.

Rat *Cyp2a* family includes *Cyp2a1*, *2a2* and *2a3*. Rat *Cyp2a1* (female dominant) and *Cyp2a2* (male dominant) are expressed in the liver (2%). In contrast, CYP2A3 is not expressed in the rat liver and is constitutively expressed in the esophagus, lung and nasal epithelium, but not in the liver, intestine, and kidney. The rat cyp2a1/2 show about 60% homology in amino acid sequence to human CYP2A6. In contrast to human, several endogenous steroids are good substrates for rat Cyp2a1/2. Rat Cyp2a1 catalyzes 7α-hydroxylation of testosterone and Cyp2a2 is involved in testosterone 15α- and 7α-hydroxylation. Mouse *Cyp2a* family includes *Cyp2a4*, *2a5*, *2a12*, and *2a22*. Mouse Cyp2a5 resembles the human orthologue in catalyzing 7-hydroxylation of coumarin. Dogs have CYP2A13 and 2A25, rabbits express CYP2A10 and 2A11, and monkeys contain CYP2A23 and 2A24. These CYP2A/2a members from various species demonstrate different substrate specificity, tissue expression, and inhibition profiles.

The mammalian CYP3 and 5 families belong to clan 3 as insect CYP6 and 9 families, mollusk CYP30 family, and *C. elegans* CYP13 and 25 families. The CYP3 family contains 6 subfamilies, CYP3A, 3B, 3C, 3D, 3E, and 3F [65]. CYP3B, 3C, 3D and 3F are fish-specific. CYP3A exists in all classes of vertebrates, comprising amphibian-, bird-, and mammal-specific clades. Members of the CYP3A subfamily appear to have been duplicated independently. The CYP3 clan contains vertebrate CYP3 and CYP5 families, insect CYP6 and 9 families, the clam CYP30 family and *C. elegans* CYP25 and 13 families, as well as other named or unnamed families from various species. The common ancestor of the CYP3 clan was likely to occur 800–1100 Mya. The ancestral vertebrates had a single *CYP3A* gene that underwent independent diversification in bony fishes, reptiles and mammals [65]. The ancestral amniota genome had two *CYP3A* genes, one of which was lost at the origin of eutherian mammals, and the other underwent gene translocation. Most *CYP3A* genes in mammals resulted from recent gene duplication events. For example, there were two *Cyp3a* gene duplication events in rodents, while rapid evolutionary changes occurred in primates and the expansion of the CYP3A subfamily significantly differed among species.

The CYP4 family includes at least 127 subfamilies (4A–4H, 4J–4N, 4P–4Z, 4AA–4AF, 4AH, 4AJ–4AN, 4AP–4AZ, 4BA–4BH, 4BJ–4BN, 4BP–4BZ, 4CA–4CH, 4CJ–4CN, 4CP–4CZ, 4DA–4DH, 4DJ–4DN, 4DP–4DZ, and 4FA). CYP4A and 4X/Z are specific to mammals, whereas 4B, 4F, and 4V are common to all vertebrates. The members of the 4F subfamily have formed several species-specific clusters, except CYP4F22. CYP4AA, 4BA, 4CA, 4FA, etc., are all found in insects only. The human CYP4 family is large with 6 subfamilies, consisting of 12 protein-coding genes and 26 pseudogenes. The 12 functional genes include *CYP4A11*, *4A22*, *4B1*, *4F2*, *4F3*, *4F8*, *4F11*, *4F12*, *4F22*, *4V2*, *4X1*, and *4Z1*. The 26 pseudogenes include *CYP4A26P*, *4A27P*, *4A43P*, *4A44P*, *4F9P*, *4F10P*, *4F23P–4F27P*, *4F29P–4F36P*, *4F44P*, *4F45P*, *4F59P–4F62P*, and *4Z2P*. The 21 *CYP4F* pseudogenes are dispersed in chromosomes 1, 2, 8, 9, 13, 18, 19, and 21.

The CYP5 family has only a single member in each species, namely CYP5A1, which is also known as thromboxane A synthase 1 (TBXAS). The *TBXAS1* gene is conserved in the dog, cow, mouse, rat, chicken, zebrafish, fruit fly, *A. thaliana*, rice, and frog; it has an orthologous gene in 151 organisms. The CYP6 family is large and present in insects. CYP6 family contains at least 162 subfamilies. These include CYP6A–6H, 6J–6N, 6P–6Z, 6AA–6AH, 6AJ–6AZ, 6BA–6BH, 6BJ–6BN, 6BP–6BZ, 6CA–6CH, 6CJ–6CN, 6CP–6CZ, 6DA–6DH, 6DJ–6DN, 6DP–6DZ, 6EA–6EH, 6EJ–6EN, 6EP–6EZ, 6FA–6FH, 6FJ–6FN, and 6FP–6FU.

Both CYP7 and 19 (aromatase) families are chordate-specific, but they are extremely sequence divergent from other CYP clans, suggesting that they are either rapidly evolving or that they may be much older than the chordate line [10]. CYP7 may have diverged from a CYP39 precursor. CYP7 family contain 7A, 7B, 7C, and 7D subfamilies. CYP7A and 7B are present in animals, while CYP7C and 7D are fish-specific. CYP7A1, a cholesterol 7α-hydroxylase, is conserved in the human, chimpanzee, Rhesus monkey, dog, cow, mouse, rat, chicken, zebrafish, and frog; 157 organisms have orthologs with human CYP7A1. CYP7B1 is a 25-hydroxycholesterol 7α-hydroxylase which is expressed from frog to human.

The CYP8 family contains only CYP8A and 8B subfamilies. Both CYP8A1 and 8B1 are conserved from frog and fish to human. CYP8B2–8B4 are present in the fish only. CYP9 family is present in insects only. This family contains at least 48 subfamilies, including CYP9A–H, 9J–9N, 9P–9Z, 9AB–9AH, 9AJ–9AN, and 9AP–9AZ subfamilies. CYP10 family present in insects only contains CYP10A, 10B and 10C subfamilies.

CYP11 family has CYP11A, 11B and 11C subfamilies. CYP11A and 11B are conserved from the frog to human, while CYP11C is fish-specific. CYP12 family is present in insects only, consisting of 13 subfamilies including CYP12A–12H and 12J–12N. CYP13 family has only one subfamily CYP13B present in *C. elegans*. CYP14 family is present in *C. elegans*, with only one CYP14A subfamily. CYP20A1 does not show any catalytic activity toward a number of potential steroids and biogenic amines. CYP21A2 is required for the synthesis of steroid hormones including cortisol and aldosterone. CYP21A2 is an important enzyme that is required for the glucocorticoids and mineralocorticoids synthesis. CYP24A1 involves in deactivation of the active form of vitamin D_3_ through the C24 oxidation pathway. 24-Hydroxylcholesterol is a better substrate for CYP46A1 than cholesterol. Mutations of *CYP46A1* may be associated with Alzheimer’s disease. CYP51A1 catalyzes a complex 14α-demethylation reaction with the aid of cytochrome P450 reductase. CYP51A1 in mammals is also responsible for production of the follicular fluid meiosis-activating sterol. Mutations of *CYP51A1* are associated with pregnancy pathologies.

Identification of the paralogs and orthologs of human CYPs has important implications in drug discovery and toxicological studies. A panel of species including mouse, rat, rabbit, dog, etc. are commonly used in these fields. However, there are remarkable species-specific differences in CYP ortholog expression and tissue distribution patterns, substrate specificity and activities, and inhibitor profiles. This may make the extrapolation form animal models to humans difficult or inaccurate. For example, rodents are not proper models for human CYP2A6 studies due to species-specific CYP2A6 ortholog expression patterns and substrate specificity and activities. Rats have little or no coumarin 7-hydroxylation activity and it is the ortholog Cyp2a1 catalyzes 3,4-epoxidation of coumarin [66]. Human CYP2A6 converts nicotine to cotinine, but rat Cyp2b1, not Cyp2a1, catalyzes this reaction. Mice have 4 orthologs of human *CYP2A6*: *Cyp2a4*, *2a5*, *2a12*, and *2a22*. The choice of proper animal models for human CYP2D6 studies is also difficult because there are significant differences between rodents and humans in the structure and number of active *CYP2D* genes in the *CYP2D/Cyp2d* locus. The mouse has nine different active *Cyp2d* genes [47] and the rat harbors six functional *Cyp2D* genes, whereas the human carries only one (CYP2D6), which indeed is absent from 7% of the Caucasian population. Mice contain *Cyp2d9*-*2d13*, *2d22*, *2d26*, *2d34*, and *2d40* and seven pseudogenes (*2d32p*, *2d33p*, *2d35p*-*2d39p* and *2d41p*) [47,67,68,69,70]. All mouse Cyp2ds have high amino acid sequence identity (65%–75%) compared with human CYP2D6. Cyp2d22 has been suggested to be the functional ortholog of human CYP2D6. Five *Cyp2d* genes, namely *Cyp2d1-2d5*, have been identified in rats by genomic analysis [47,70]. Rat Cyp2d5 has >95% similarities in amino acid sequence to Cyp2d1 and Cyp2d4. Rat *Cyp2d3*, but not *Cyp2d1*, *2d2* or *2d4*, is the homolog of human, chimpanzee, Rhesus monkey and chicken *CYP2D6* and frog *Cyp2d6* and *2d20*. In NCBI Gene database and the assembly Cavpor3.0, the guinea pig genome contains 4 active *Cyp2d* member including *Cyp2d6*, *2d16*, *2d17* and *2d27* and 1 pseudogene (*Cyp2d3p*). The rabbit genome *Cyp2d* locus contain 4 *Cyp2d* members, *Cyp2d24–2d17–2d23–2d4-ps*. Except *Cyp2d4-ps*, other three genes are functionally active. Thus, caution should be taken when extrapolating the results involving CYP2D studies from animals to humans.

In summary, the identification of orthologs is a central problem in the field of comparative genomics and phylogenetic analysis and accurate prediction of the orthologs and paralogs of human *CYP* genes is fundamental to understand the evolutionary relationships and functional implications of this superfamily of important enzymes that are involved in the biotransformation of a large number of therapeutic drugs, environmental compounds and endogenous substances. The delineation of the human *CYP* orthologs in other species also has important implications in drug discovery and biomedical research when animal models are widely used. On the other hand, phylogeny-based orthologous relationships may not be enough to describe the evolutionary and functional relationships of human *CYPs*, other factors such as the protein 3D structures and protein interaction networks should be taken into account.

## 4. Methods

### 4.1. Human Cytochrome P450s (CYPs) in Current Human Assembly GRCh38.p6 and Sequence Alignment of Human CYPs

The current human genome assembly is GRCh38.p6 (GenBank assembly accession: GCA_000001405.21) which was released on 21 December 2015 by the Genome Reference Consortium. GRCh38.p2 was released in December 2014, which has offered a data set based on the *Homo sapiens* high-coverage assembly GRCh38 released by the Genome Reference Consortium in December 2013. In this assembly, there are 20,300 coding genes with 198,457 transcripts, 25,159 non-coding genes, and 14,424 pseudogenes. Identification of *CYPs* in any organisms is critical based on featured motifs in their protein sequences. For example, almost all CYPs carry two CYP signature motifs: one is “FXXGXRXCXG” (also known as “CXG”) located in the heme-binding domain and another one is the “EXXR” motif located in helix K. The genome of human beings carries 57 functional *CYP* genes and 58 pseudogenes as well. Previously, we thought *CYP2D7* as a pseudogene, but now it has been considered as a functional gene in humans by HGNC (ID: 2624), UniProtKB (A0A087X1C5), Ensembl 84 (ENSG00000205702), and GeneCards 4.1.1 (GCID: GC22M042140). However, NCBI GenBank (ID: 1564) still lists *CYP2D7* as a pseudogene.

The primary protein sequences of 57 functional CYPs present in humans were retrieved from the UniProtKB/Swiss-Prot database (http://www.uniprot.org/). Multiple sequence alignment of the human CYPs was carried out using Clustal W v2.0 (http://www.clustal.org) with all parameters set as default. The phylogenic tree of human *CYPs* was also built up in order to deduce the evolutionary relationships among these human *CYP* sequences. In addition, the MEME program version 4.10.1 (http://meme-suite.org) was employed to identify characteristic motifs present in human CYPs.

### 4.2. Computational Identification of the Paralogs, Homologs, and Orthologs of Human CYPs

Paralogs are defined as homologous genes in one species which arise from a gene duplication event in the genome [71,72,73]. Different from orthologous genes, a paralog is a novel gene with new function, although the new function is always associated with the biological role of the ancestral gene. If the mutations produce stop codons or frameshift, paralogs may eventually become pseudogenes. In GeneCards 4.1.1 (Table 2), GeneDecks is used to predict functional paralogs based on combinatorial similarity of attributes [74]. Paralogs in GeneCards are from HomoloGene, Ensembl, and SIMAP (http://liferay.csb.univie.ac.at), and pseudogenes from Pseudogene.org. In Ensembl release 84, paralogs are identified by a multi-step approach where the maximum likelihood phylogenetic gene trees are built [75]. In Ensembl, paralogous genes are defined as those for which the most common ancestor node is a duplication event (see below).

A homolog is a gene similar in structure and evolutionary origin to a gene in another species [73,76,77]. The term “homolog” may apply to the evolutionary relationship between genes split by speciation event (i.e., ortholog), or to the one between genes arising from a duplication event (i.e., paralog). As such, orthologous genes are defined as homologous genes separated by a speciation event in the genome during evolution, and these genes largely retain a similar function to that of the ancestral gene [41,78,79,80,81]. Speciation, an evolutionary process, gives rise to new species that can live in a new way from the parent species. There are four geographic types of speciation in nature, based on the extent to which speciating populations are separated from one another, namely allopatric, peripatric, parapatric, and sympatric. Speciation has obtained some barriers to genetic exchange with the parent species. Orthologous genes generally show ≥70% of DNA or protein sequence identity. Homologous genes often maintain the function of their ancestral gene through a speciation event, although genetic variations may arise after the new species arises. Therefore, functions may be lost or gained when comparing a pair of orthologs. There are difficulties in confirming the exact ancestry of homologous genes in various organisms due to the frequent occurrence of gene duplication and genome rearrangement. Phylogenetic analysis of the gene lineage always provides evidence whether two similar genes from distinct organisms are orthologous.

In NCBI, HomoloGene 68 released in April 2014 was used as an automated system for building up putative homologous groups based on the complete genomes of 21 eukaryotic species (Table 2 and Table S1). The protein sequences are compared to one another using the blastp (protein-protein BLAST) program and then are matched up to give rise to groups, using a tree developed from sequence similarity to guide the constructing process. During the process, closely related organisms are matched up first, and then more organisms are added as the tree is traversed toward the root. Thereafter, the protein sequence alignments are mapped back to their corresponding DNA sequences by which distance metrics such as molecular distance and the nonsynonymous (Ka) to synonymous (Ks) ratio (Ka/Ks) can be determined. Sequences are aligned using synteny when appropriate. In a bipartite matching, residual sequences from other organisms are aligned using an algorithm that will force to maximize the global score. Cutoff values on bits per position and Ks values are predetermined to avoid incorrect grouping of “unlikely” orthologs. The cutoff values are determined using the score distribution for a given group of organisms. In addition, paralogous genes are also found via matching sequences.

We further employed 8 online databases to identify the orthologs of human *CYP* genes: NCBI, Ensembl Compara, GeneCards, OMA (“Orthologous MAtrix”) Browser, PATHER, TreeFam, EggNOG, and Roundup (Table 2). In NCBI, the Annotation Pipeline method is used to identify the orthologous genes in selected vertebrae genomes [80]. In this approach developed by NCBI, a process flow has been created using the vertebrate RefSeq sequences to investigate the genes, protein sequence conservation, and annotation consistency. Briefly, the NCBI approach discovers sets of comparable proteins present in various vertebrates, including orthologs and similar proteins among alternatively splicing products. This protocol combines sequence, protein-coding regions, and functional annotation via identification of featured conserved domains to discover conservation from multiple levels [80]. Protein sequence alignments are efficiently conducted using BLAST. As such, mRNA transcript sequences and annotated protein-coding regions of the genes can be mapped onto their respective protein sequence alignments to identify splice conservation across different orthologous genes. The RefSeq database can identify sets of orthologs via best hits to corresponding Swiss-Prot proteins as sets of potential homologous genes and the orthology is finally confirmed through local synteny. In NCBI, the genomes of 187 vertebrate species including 22 primates, 16 rodents, 17 even-toed ungulates and whales (Cetartiodactyla), 36 other mammals, 58 birds, 27 fishes, and 11 other vertebrates, 50 insects, 15 other invertebrates, and 39 plants have been annotated completely (Table S1). These species have been included to identify orthologs of human *CYP* genes. Presently, all the model species and organisms from the Homologene database are included for ortholog identification in GeneCards version 4.1.1 released in March 2016 (Table S1).

In Ensembl 84, released in March 2016, orthologous and paralogous genes are predicted using the TreeBeST protocol that will finally build up maximum likelihood phylogenetic trees of 68 chordates (Table S1) [75,81,82,83]. The resultant phylogenetic trees merged with their species tree carry internal nodes that have been annotated to discern duplication or speciation events. To begin the prediction process, the TreeBeST protocol will load a representative translated protein of each gene from species used in Ensembl. From each gene tree, gene pairwise relations of orthologs and paralogs are inferred, and orthologs are finally verified using the model species and organisms of chordates in Ensembl (Table S1). GeneCards 4.1.1 contains orthologs from several databases including HomoloGene, Ensembl Pan Taxonomic Compara, SGD, MGI Flybase, WormBase (through Ensembl), and euGenes. The species from Ensembl Pan Taxonomic Compara are selected to create a diverse panel of taxa including model organisms and species of interest. In addition, all available species from Homologene are incorporated in GeneCards 4.1.1.

OMA Browser is a large database that can be used to infer orthologous genes amongst species with known complete genomes and translated proteomes [84] (http://omabrowser.org). To calculate homologous sequences, all-against-all Smith-Waterman alignments are performed and significant matches are kept. The orthologous genes are discovered based on evolutionary distances, in view of distance inference uncertainty and possible differential gene losses [85]. In OMA, homologous genes are defined as pairs of homologous genes that have commenced diverging through speciation events between the progenitor genomes and then merged back into the same genome by hybridization. Thus, homologs can be considered as “orthologs between subgenomes”. OMA Browser covers all domains of life including 226 species of Eukaryota, 1353 species of Bacteria, and 127 species of Archaea. In the current release, (release 17), the database includes a total of 883,176 OMA groups and 8,798,758 proteins. Notably, OMA now has made 442,376,477 function annotations for a total of 7,947,728 proteins.

PANTHER is also used to predict the orthologs of human *CYP* genes via analyses of evolutionary relationships among 104 model organisms and inference of gene function using a total of 41,603 GO terms (Table S1) [86,87] (http://pantherdb.org/). In PANTHER, the phylogenetic trees are constructed to exhibit gene family evolution with incorporation of evolutionary events (e.g., speciation and duplication) [86]. The current version 10.0 released in May 2015 contains a total of 11,928 protein families which are further divided into 83,190 functionally distinct subfamilies.

The TreeFam database carry phylogenetic trees inferred from the genomes of animals and thus perform predictions of orthologous and paralogous genes (http://www.treefam.org/). The current release (release 9) contains 109 species (Table S1) and 15,736 gene families. In TreeFam, a gene family is defined as a group of genes arising from speciation of single-metazoan animals. TreeFam uses protein identifiers from Ensembl, Ensembl Genomes, Wormbase, and JGI (http://genome.jgi.doe.gov). In EggNOG (“evolutionary genealogy of genes: Non-supervised Orthologous Groups”) 4.1, orthologous genes are automatically inferred by splitting species space into “core” and “periphery” species [88] (http://eggnogdb.embl.de). The core species are critical for finding orthologous genes using the strict triangular criterion. Most of the phylogenetic trees in EggNOG are reconstructed using a strategy similar to the one described by Huerta-Cepas et al. [89], which uses a combination of multiple sequence aligners, alignment trimming techniques, model testing, and maximum likelihood inference. The model yielding the best maximum likelihood value is applied to infer a final tree with Phyml and full Maximum Likelihood optimization. The current 4.1 release contains 2031 organisms, 9.6 millions of proteins, and 190,000 orthologous groups. Finally, RoundUp 2.0 is a large-scale on-line database of orthologous genes (http://roundup.hms.harvard.edu). The orthologous genes across species are identified using the Reciprocal Smallest Distance (RSD) algorithm [90]. This algorithm used is able to discover more and more precise orthologous genes than reciprocal best blast hits and assigns each orthologous gene a score according to its maximum likelihood evolutionary distance. Roundup contains more than 1800 genomes that are from 226 eukaryota, 1447 bacteria, 113 archaea, and 21 viruses.

## Figures and Tables

**Table 1:** A list of 57 human functional *CYP* genes and their corresponding paralogs based on Ensembl 84 and GeneCards 4.1.1.

Gene	Chromosomal Location	Substrates/Function	Number of Amino Acids	Paralogs by Ensembl 84	Paralogs by GeneCards 4.1.1
*CYP1A1*	15q24.1	Drugs, procarcinogens, steroids, and fatty acids	512	*CYP1A/1B*, *17A1* and *21A1*	
*CYP1A2*	15q24.1	Drugs, fatty acids, and steroids	516		
*CYP1B1*	2p22.2	Drugs, procarcinogens, steroids, and fatty acids	543		
*CYP2A6*	19q13.2	Drugs and steroids	494	*CYP2* members	*CYP2* members excluding *CYP2D7*
*CYP2A7*	19q13.2	Unknown (orphan)	494		
*CYP2A13*	19q13.2	Drugs and other xenobiotics	494		
*CYP2B6*	19q13.2	Drugs, steroids and fatty acids	491		
*CYP2C8*	10q23.33	Drugs, steroids and fatty acids	490		
*CYP2C9*	10q24	Drugs, steroids and fatty acids	490		
*CYP2C18*	10q24	Drugs, steroids and fatty acids	490		
*CYP2C19*	10q24.1-q24.3	Drugs	490		
*CYP2D6*	22q13.1	Drugs	497		
*CYP2E1*	10q26.3	Drugs, ethanol, and procarcinogens	493		
*CYP2F1*	19q13.2	Drugs and coumarins	491		
*CYP2J2*	1p31.3-p31.2	Fatty acid (e.g., AA)	502		
*CYP2R1*	11p15.2	Vitamin D	501		
*CYP2S1*	19q13.1	Xenobiotics	504		
*CYP2U1*	4q25	AA, DHEA, and long chain fatty acids	544		
*CYP2W1*	7p22.3	Unknown	490		
*CYP3A4*	7q21.1	Drugs, steroids and fatty acids	503	*CYP3*, *4*, *5* and *46* members and *CYP3A7-3A51P*	*CYP3*, *4*, and *5* members
*CYP3A5*	7q21.1	Drugs, steroids and fatty acids	502		
*CYP3A7*	7q21-q22.1	Drugs, steroids and fatty acids	503		
*CYP3A43*	7q21.1	Low level of testosterone 6β-hydroxylase activity	503		
*CYP4A11*	1p33	Medium-chain fatty acids such as laurate and myristate	519		
*CYP4A22*	1p33	Unknown (orphan)	519		
*CYP4B1*	1p33	Xenobiotics, steroids and fatty acids	511		
*CYP4F2*	p13.12	Eicosanoids	520		
*CYP4F3*	19p13.2	Eicosanoids (e.g., LTB_4_)	520		
*CYP4F8*	19p13.1	Eicosanoids	520		
*CYP4F22*	19p13.1	Unknown (orphan)	524		
*CYP4F22*	19p13.1	Fatty acids	524		
*CYP4F22*	19p13.12	Unknown (orphan)	531		
*CYP4V2*	4q35.2	Unknown (orphan)	525		
*CYP4X1*	1p33	Unknown (orphan)	509		
*CYP4Z1*	1p33	Flavoprotein hydroxylation	505		
*CYP5A1*	7q34-q35	Thromboxane synthesis	534		
*CYP7A1*	8q11-q12	Cholesterol	504	*CYP7*, *8* and *39* members	
*CYP7B1*	8q21.3	Cholesterol	506		
*CYP8A1*	20q13.13	Isomerisation of PGH_2_ to prostacyclin	500		
*CYP8B1*	3p22-p21.3	Steroids	501		
*CYP11A1*	15q23-q24	Side-chain cleavage of cholesterol pregnenolone	521	*CYP24* and *27* members	*CYP11*, *19*, *24*, *27* and *46* members
*CYP11B1*	8q21	Steroids	503		
*CYP11B2*	8q21-q22	Steroids, especially production of aldosterone	503		
*CYP17A1*	10q24.3	Steroid metabolism, especially the conversion of pregnenolone and progesterone	508	*CYP1* and *21 members*	*CYP1* and *21* members
*CYP19A1*	15q21.1	Steroid metabolism, formation of aromatic C18 estrogens and C19 androgens	503	No paralog	*CYP11*, *24*, *27* and *46* members
*CYP20A1*	2q33.2	Unknown (orphan)	462	No paralog	
*CYP21A2*	6p21.3	21-hydroxylation of steroids; required for adrenal synthesis of mineralocorticoids and glucocorticoids	495	*CYP1* and *17 members*	*CYP1A1, 1A2* and *17A1*
*CYP24A1*	20q13	Vitamin D hydroxylation	514	*CYP11* and *27* members	*CYP11*, *19*, *27* and *46* members
*CYP26A1*	10q23-q24	Retinoic acid metabolism	497	*CYP26* and *51* members	
*CYP26B1*	2p13.2	Retinoic acid metabolism	512		
*CYP26C1*	10q23.33	Retinoic acid metabolism	522		
*CYP27A1*	2q35	Steroid metabolism, catalyzing first step in oxidation of side-chain of sterol intermediates	531	*CYP11*, *24*, and *27* members	*CYP11*, *19*, *24*, and *46* members
*CYP27B1*	12q14.1	Vitamin D metabolism	508		
*CYP27C1*	2q14.3	Unknown (orphan)	372		
*CYP39A1*	6p21.1-p11.2	Cholesterol	469	*CYP7* and *8* members	
*CYP46A1*	14q32.1	Cholesterol	500	*CYP3*, *4*, and *5* members and *CYP3A7-3A51P*	*CYP11*, *19*, *24*, and *27* members
*CYP51A1*	7q21.2-	Sterols	509	*CYP26* members	

**Table 2:** Databases and programs used to predict the orthologs of human cytochrome P450 (*CYP*) genes.

Database	URL	Current Release	Primary Application	Number of Organisms	Taxonomic Range	Update Frequency
NCBI	http://www.ncbi.nlm.nih.gov/	RefSeq release 75 (released on 14 March 2016)	NCBI’s genome annotation pipeline	862 mammalian vertebrates and 3145 other vertebrates (RefSeq covers 58,776 organisms)	Vertebrates	Daily
Ensembl Compara	http://useast.ensembl.org/index.html	Release 84 (released in March 2016)	To build phylogenetic trees across the whole set of protein-coding genes with one pipeline	68 chordates plus 240 others	All domains of life	Quarterly
GeneCards	http://www.genecards.org/	Version 4.1.1 (released on 13 March 2016)	To provide comprehensive, user-friendly information on all annotated and predicted human genes (39,629 HGNC approved, 21,976 protein-coding genes, 104,578 RNA genes, and 16,329 pseudogenes)	58	All domains of life	Yearly
OMA	http://omabrowser.org/oma/home/	Release 17 (released in September 2014)	To identify orthologous genes via massive cross-comparison of complete genomes	1706 (226 Eukaryota, 1353 Bacteria, and 127 Archaea)	All domains of life	Twice per year
PANTHER	http://pantherdb.org/	Version 10.0 (released in 25 April 2015)	Inference of gene function using GO terms and evolutional relationships of genes among organisms (11,928 protein families, divided into 83,190 functionally distinct protein subfamilies)	104	All domains of life	Yearly
TreeFam	http://www.treefam.org	Release 9 (released on 3 May 2013)	Phylogenetic tree construction and providing orthology/parology predictions as well the evolutionary history of genes	109 (vs. 79 in TreeFam 8)	Metazoans + model eukaryotes	Once every 2 years
EggNOG	http://eggnogdb.embl.de/#/app/home	4.1 (released on 5 May 2015)	A database of orthologous groups and functional annotation	2031	All domains of life	Once every 3–4 years
RoundUp	http://roundup.hms.harvard.edu/	2.0 (released in January 2012)	An online database of gene orthologs for over 1800 genomes	1807	All domains of life	2–4 times per year

## Supplementary Materials

Supplementary materials can be found at http://www.mdpi.com/1422-0067/17/7/1020/s1.
